# Optimal Self-Induced Stochastic Resonance in Multiplex Neural Networks: Electrical vs. Chemical Synapses

**DOI:** 10.3389/fncom.2020.00062

**Published:** 2020-08-07

**Authors:** Marius E. Yamakou, Poul G. Hjorth, Erik A. Martens

**Affiliations:** ^1^Max-Planck-Institut für Mathematik in den Naturwissenschaften, Leipzig, Germany; ^2^Department of Applied Mathematics and Computer Science, Technical University of Denmark, Lyngby, Denmark; ^3^Department of Biomedical Science, University of Copenhagen, Copenhagen, Denmark; ^4^Centre for Translational Neuromedicine, University of Copenhagen, Copenhagen, Denmark

**Keywords:** optimization, self-induced stochastic resonance, synapses, multiplex neural network, community structure

## Abstract

Electrical and chemical synapses shape the dynamics of neural networks, and their functional roles in information processing have been a longstanding question in neurobiology. In this paper, we investigate the role of synapses on the optimization of the phenomenon of self-induced stochastic resonance in a delayed multiplex neural network by using analytical and numerical methods. We consider a two-layer multiplex network in which, at the intra-layer level, neurons are coupled either by electrical synapses or by inhibitory chemical synapses. For each isolated layer, computations indicate that weaker electrical and chemical synaptic couplings are better optimizers of self-induced stochastic resonance. In addition, regardless of the synaptic strengths, shorter electrical synaptic delays are found to be better optimizers of the phenomenon than shorter chemical synaptic delays, while longer chemical synaptic delays are better optimizers than longer electrical synaptic delays; in both cases, the poorer optimizers are, in fact, worst. It is found that electrical, inhibitory, or excitatory chemical multiplexing of the two layers having only electrical synapses at the intra-layer levels can each optimize the phenomenon. Additionally, only excitatory chemical multiplexing of the two layers having only inhibitory chemical synapses at the intra-layer levels can optimize the phenomenon. These results may guide experiments aimed at establishing or confirming to the mechanism of self-induced stochastic resonance in networks of artificial neural circuits as well as in real biological neural networks.

## 1. Introduction

Noise is an inherent part of neuronal dynamics, and its effects can be observed experimentally in neuronal activity at different spatiotemporal scales, e.g., at the level of ion channels, neuronal membrane potentials, local field potentials, and electroencephalographic or magnetoencephalographic measurements (Guo et al., 2018). While noise is mostly undesirable in many systems, it is now widely accepted that its presence is crucial to the proper functioning of neurons in terms of their information processing capabilities.

Some mechanisms for optimal information processing are provided via the well-known and extensively studied phenomena of stochastic resonance (SR) (Benzi et al., 1981; Longtin, 1993; Gammaitoni et al., 1998; Lindner et al., 2004; Zhang et al., 2015) and coherence resonance (CR) (Hu and MacDonald, 1993; Neiman et al., 1997; Pikovsky and Kurths, 1997; Lindner and Schimansky-Geier, 1999; Lindner et al., 2004; Beato et al., 2007; Hizanidis and Schöll, 2008; Liu et al., 2010; Bing et al., 2011; Gu et al., 2011) or via the lesser-known phenomenon of self-induced stochastic resonance (SISR) (Freidlin, 2001; Muratov et al., 2005; DeVille and Vanden-Eijnden, 2007; DeVille et al., 2007; Yamakou and Jost, 2017, 2018) whose mechanism remains to be confirmed experimentally in real neural systems. Although these noise-induced phenomena may exhibit similar dynamical behaviors, each of them has different dynamical preconditions and emergent mechanisms and may therefore play different functional roles in information processing. For further details behind the mechanisms of SR and CR, we refer the reader to references given above. We also note that the control of SR and CR in neural networks has attracted a lot of attention. In particular, it has been shown that hybrid synapses and autapses (i.e., those characterized by both electrical and chemical coupling) could be effectively used to control SR and CR (Yilmaz et al., 2013, 2016).

In this paper, we focus on self-induced stochastic resonance (SISR). SISR can occur when a multiple timescale excitable dynamical system is driven by vanishingly small noise. During SISR, the escape time of trajectories from one attracting region in phase space to another is distributed exponentially, and the associated transition frequency is governed by an activation energy. Suppose the system describing the neuron is placed out of equilibrium, and its activation energy decreases monotonically as the neuron relaxes slowly to a stable quiescent state (fixed point); then, at a specific instant during the relaxation, the timescale of escape events and the timescale of relaxation match, and the neuron almost surely fires at this point. If this activation brings the neuron back out-of-equilibrium, the relaxation stage can start over again, and the scenario repeats itself indefinitely, leading to a cyclic coherent spiking of the neuron which cannot occur without noise. SISR essentially depends on (i) strong timescale separation between the dynamical variables; (ii) vanishingly small noise amplitude; (iii) a monotonic activation energy barrier; (iv) and, most importantly, the periodic matching of the slow timescale of neuron's dynamics to the timescale characteristic to the noise. Thus, compared to CR and SR, the conditions to be met for observing SISR are more subtle: Like CR, SISR does not require an external periodic signal as in SR. Remarkably, unlike CR, SISR does not require the neuron's parameters be close to the bifurcation thresholds, making it more robust to parameter tuning than CR. Moreover, unlike both SR and CR, SISR requires a strong timescale separation between the neuron's dynamical variables.

The mechanism behind SISR suggests that, in an excitable neuron, the level of noise embedded in the neuron's synaptic input may be decoded into a (quasi-) deterministic and coherent signal. To exemplify, in a network of neurons in a quiescent state (without any activity), the action of a sufficiently weak synaptic noise amplitude could occasionally generate a spike in each neuron. These spikes will have random phases so that their total input on each individual neuron may average to a stationary random signal of low intensity. If the noise amplitude suddenly increases due to a change in the synaptic input, the neurons may switch to the noise-assisted oscillatory mode. This can further increase the effective noise amplitude so that the oscillatory mode may persist even after the disturbance is removed and the entire neural network in a dormant state may wake up from the outside rattle. The phenomenon of SISR in neural networks could therefore play important functional roles in the regulation of the Sleep-wake transition (Patriarca et al., 2012; Booth and Behn, 2014; Pereda, 2014).

Communication between neurons occurs through synaptic interactions. Two main types of synapses may be identified in neural networks, electrical synapses and chemical synapses (Pereda, 2014). The corresponding functional form of the bidirectional interaction mediated by the electrical synapses is defined as the difference between the membrane potentials of two adjacent neurons, thereby making the coupling mediated by electrical synapses to be local. Meanwhile, chemical synaptic interaction always takes place unidirectionally, with the signal conveyed chemically via neurotransmitter molecules through the synapses, thereby making chemical synaptic couplings nonlocal. The functional form of the chemical synaptic interaction is considered as a nonlinear sigmoidal input-output function (Greengard, 2001). Moreover, chemical synapses can be inhibitory or excitatory. When an inhibitory pre-synaptic neuron spikes, the post-synapses neuron connected to it is prevented from spiking. When an excitatory neuron spikes, it induces the post-synaptic neuron to spike. In real biological neurons, the distance between pre- and post-synaptic ends is approximately 3.5 nm in electrical synapses, and it is comparatively large, nearly 20–40 nm (Hormuzdi et al., 2004), in chemical synapses. Distances between pre- and post-synaptic ends induce time delays in neural networks with the time delays of electrical synapses being generally shorter than those of chemical synapses.

It is well-known from magnetic resonance imaging that neural networks may exhibit several types of coupling schemes: neurons coupled via electrical synapses only; neurons coupled via chemical synapses only; and neurons coupled by both electrical and chemical synapses—so-called hybrid synapses (Galarreta and Hestrin, 1999, 2001; Gibson et al., 1999; Connors and Long, 2004; Hestrin and Galarreta, 2005; Yilmaz et al., 2013; Bera et al., 2019; Majhi et al., 2019). Moreover, multiplex networks of neurons can be formed from different network layers depending on their connectivity through a chemical link or by an ionic channel. In brain networks, different regions can be seen connected by functional and structural neural networks (Pisarchik et al., 2014; De Domenico, 2017; Andreev et al., 2018). In a multiplex network, each type of interaction between the nodes is described by a single layer network and the different layers of networks describe the different modes of interaction. Multilayer networks (Pisarchik et al., 2014) open up new possibilities of optimization, allowing to regulate neural information processing by means of the interplay between the neurons' dynamics and multiplexing (Crofts et al., 2016; Battiston et al., 2017). Optimization based on multiplexing could have many advantages. In particular, the coherent spiking activity of one layer (induced for example by SISR) can be optimized by adjusting the parameters of another layer. This is important from the point of view of engineering and brain surgery since it is not always possible to directly access the desired layer, though the network with which this layer is multiplexed may be accessible and adaptable.

Several studies have shown that multiplex networks can generate patterns with significant differences from those observed in single-layer networks (Kouvaris et al., 2015; Majhi et al., 2016, 2017; Berner et al., 2020). Their use in the optimization and control of dynamical behaviors have therefore attracted much attention recently. The multiplexing of networks has been shown to control many dynamical behaviors in neural networks, including synchronization (Gambuzza et al., 2015; Singh et al., 2015; Andrzejak et al., 2017; Leyva et al., 2017; Zhang et al., 2017), pattern formation (Kouvaris et al., 2015; Ghosh and Jalan, 2016; Ghosh et al., 2016, 2018; Maksimenko et al., 2016; Bera et al., 2017; Bukh et al., 2017), solitary waves (Mikhaylenko et al., 2019), and chimera states (Panaggio and Abrams, 2015; Schöll, 2016; Ghosh et al., 2018, 2019; Omelchenko et al., 2018; Sawicki et al., 2019). Chimera states are synchronization patterns occurring in symmetric networks (on average), characterized by the coexistence of varying synchronization levels side-by-side. They have been shown to exist in mechanical and chemical experiments (Tinsley et al., 2012; Martens et al., 2013; Totz et al., 2017) and are thought play an important role in neural systems (Andrzejak et al., 2016; Bera et al., 2019; Majhi et al., 2019). In particular, synchronization patterns such as chimera states occur in networks with community structure where connections are all-to-all, but coupling strengths are modulated so that the inter-coupling between communities (layers) are weak/sparse compared to their intra-coupling (Abrams et al., 2008; Martens et al., 2016a,b; Bick et al., 2020)—a configuration that bears strong similarity with the multilayer structure. Chimera states in such networks are of interest as they are multistable (Martens, 2010) and thus configurable; they can in principle be employed to solve functional tasks such as computations (Bick and Martens, 2015) and routing of information (Deschle et al., 2019) in the brain. Moreover, community networks of QIF neurons exhibit synchronization patterns that have been demonstrated viable for memory storage and recall (Schmidt et al., 2018). However, the optimization of noise-induced resonance mechanisms in neural networks based on the multiplexing approach have only very recently attracted attention. The few research works investigating the optimization of CR in neural networks are those of Semenova and Zakharova (2018) and Yamakou and Jost (2019).

In Semenova and Zakharova (2018), it is shown that connecting a one-layer network exhibiting CR in a multiplex way to another one-layer network, i.e., multiplexing, allows us to control CR in the latter layer network. In particular, it is found that multiplexing induces CR in networks that do not demonstrate this phenomenon in isolation. Moreover, it has been shown that CR can be achieved even for weak multiplexing between the layers. Surprisingly, it has also been shown that the multiplex-induced CR in the layer which is deterministic in isolation can manifest itself even more strongly than the CR in the noisy layer. However, the work in Semenova and Zakharova (2018) considers only instantaneous synaptic connections, while it is well known that synaptic time delays (not negligible in neural networks) exhibit crucial effects in neural information processing.

Yamakou and Jost (2019) considered synaptic time delays and their role in optimizing CR in a layer affected by another layer via multiplexing, which already exhibits optimal CR or SISR. In an isolated layer, it was shown that shorter synaptic time delays combined with weaker synaptic strengths optimize CR. Meanwhile, in the multiplex network configurations, stronger synaptic strengths combined with shorter synaptic time delays between layers induce and optimize CR in the layer where this phenomenon is non-existent in isolation. Moreover, their numerical simulations indicate that, even at very long multiplexing time delays, weak (but not too weak) multiplexing strengths between the layers can induce and optimize CR in the layer where it is non-existent in isolation. Interestingly, it was further shown that, with the occurrence of a different resonance phenomenon (i.e., SISR) in one layer, weak multiplexing, even at very short synaptic time delays, completely fails to optimize CR in the other layer where latter phenomenon does not exist in isolation. This behavior further confirms the fact that, even though SISR and CR lead to the occurrence of the same dynamical behavior (i.e., coherent noise-induced spiking activity) in neurons in the excitable regime, they are fundamentally different in their dynamical and emergent nature (DeVille et al., 2005); in particular, SISR and CR also lead to different behaviors in multiplex networks, and they possibly therefore play different functional roles in neural information processing.

The optimization of CR in neural networks based on the multiplexing approach have so far been studied only in Semenova and Zakharova (2018) and Yamakou and Jost (2019). A study on the optimization of SISR in neural networks based on the multiplexing approach is still lacking. Moreover, in Semenova and Zakharova (2018) and Yamakou and Jost (2019), the coupling between the neurons are mediated only by electrical synapses. The role of chemical synapses in the optimization of noise-induced resonance mechanisms should be equally important. Therefore, the aim of this paper is to study the optimization of SISR based on the multiplex approach of neural networks connected through time-delayed electrical and chemical synapses. In particular, we wish to address the following main questions:

- Can SISR occurring in one layer of a multiplex network be used to optimize SISR in another layer where the phenomenon non-existent in isolation?
- What combinations of intra- and inter-layer synaptic strengths and time delays best optimize SISR?
- Which type (electrical, inhibitory, or excitatory) of synapse is best optimizer SISR within an isolated layer and in the multiplex configuration?

The rest of the paper is organized as follows: in section 2, we present the mathematical model equations, and we explain and motivate the different configurations considered. In section 3, we briefly describe the numerical methods used in simulations and analysis. In section 4, we consider an isolated single layer of neurons, coupled either by electrical synapses or chemical synapses. For both types of coupling, we analytically establish the necessary conditions in terms of noise amplitudes and timescale separation parameter that allow us to observe SISR. In section 5, we systematically investigate synaptic parameterizations that best optimize SISR in an isolated layer in which the neurons are coupled either by electrical synapses or by inhibitory chemical synapses. We will then compare the optimization of SISR by electrical and inhibitory chemical synapses. In section 6, we consider multiplexed layer networks using numerical simulations. Having identified which synaptic configurations deteriorate SISR the most in isolated layers, we use the multiplexing between a first layer, where SISR is optimal and a second layer where SISR is non-optimal (very poor or even non-existent), with the goal of optimizing SISR in the second layer. For multiplex networks, we will consider the optimization of SISR in six case scenarios: electrical, inhibitory, and excitatory multiplexing of two layers with electrical synaptic intra-connections and electrical, inhibitory, and excitatory multiplexing of two layers with inhibitory synaptic intra-connections. Finally, we summarize and conclude our findings in section 7.

## 2. Mathematical Model

**Figure 1:**
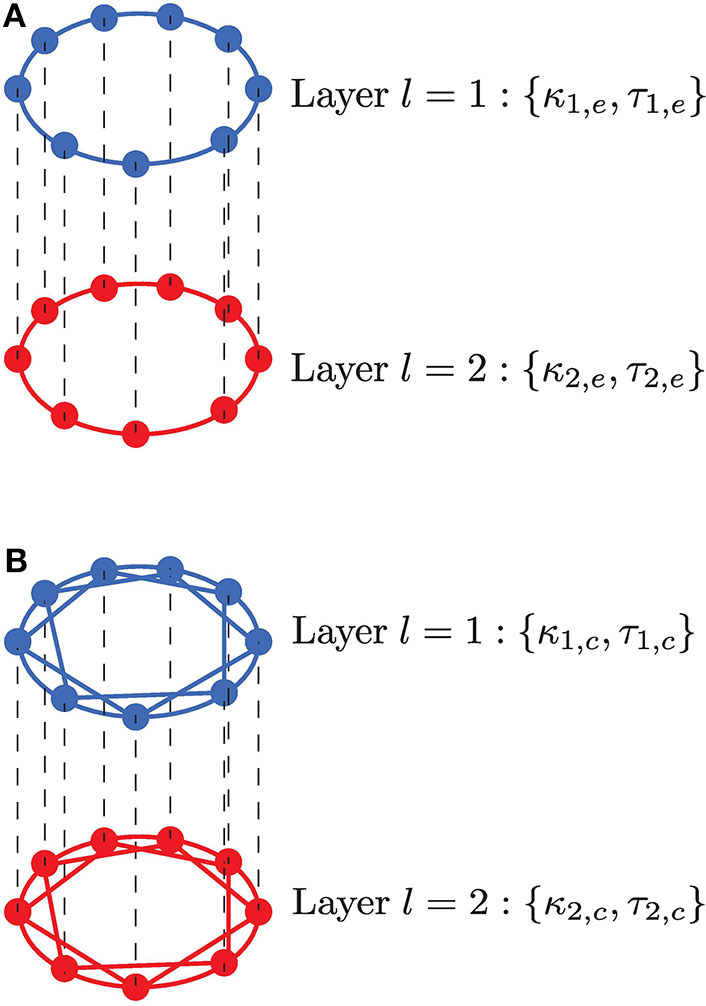
Neurons are connected in a multiplex network with two layers *l* = 1, 2. Neurons within a layer are coupled in a ring, while neurons in adjacent layers are connected only to their adjacent neurons. **(A)** Neurons within each layer are coupled in a ring and interact only with their nearest neighbors via electrical synapses (κ_1,*e*_, τ_1,*e*_, κ_2,*e*_, τ_2,*e*_); multiplexing between these layers, represented by the black vertical dashed lines, may occur via electrical (κ_*m,e*_, τ_*m,e*_) or (inhibitory or excitatory) chemical synapses (κ_*m,c*_, τ_*m,c*_). **(B)** Neurons within each layer are coupled in a ring and interact with *n*_*l,c*_ nearest neighbors via inhibitory chemical synapses (κ_1,*c*_, τ_1,*c*_, κ_2,*c*_, τ_2,*c*_); multiplexing between these layers, represented by the black vertical dashed lines, may occur via electrical (κ_*m,e*_, τ_*m,e*_) or (inhibitory or excitatory) chemical (κ_*m,c*_, τ_*m,c*_) synapses. In both scenarios, an enhanced SISR in layer *l* = 1 is used to optimize a poor or non-existent SISR in layer *l* = 2 by variation of the time-delay coupling parameters within a population (κ_1,*e*_, τ_1,*e*_, κ_2,*e*_, τ_2,*e*_, κ_1,*c*_, τ_1,*c*_, κ_2,*c*_, τ_2,*c*_) and between populations (κ_*m,e*_, τ_*m,e*_, κ_*m,c*_, τ_*m,c*_).

We consider a two-layer multiplex neuronal network in the excitable regime in the presence of synaptic noise, as illustrated in Figure 1. In our study, we consider one of the simplest network topologies—a ring network topology within layers and a multiplex network between these layers such that they contain the same number of neurons and the interaction between the layers are allowed only for replica neurons. Each layer consists of *N* identical FitzHugh-Nagumo (FHN) neurons (Hodgkin and Huxley, 1952; FitzHugh, 1961), connected in a ring by either only electrical synapses or inhibitory chemical synapses, while the inter-connections between layers can be either via electrical, inhibitory chemical synapses, or excitatory chemical synapses. It is important to point out that excitatory chemical synapses are found to induce, via time-delayed coupling bifurcations, a self-sustained spiking activity in the network of FHN neurons (each in the excitable regime) even in the complete absence of noise. We want to avoid such regimes—those in which the deterministic network can oscillate due to some time-delayed coupling induced bifurcations—as the coherent oscillations induced by SISR should be due only to the presence of noise and not because of the occurrence of bifurcations. For this reason, the excitatory chemical synapses are used in the optimization of SISR only when they do not induce oscillatory behaviors in the deterministic network, i.e., only in the multiplexing connections with carefully chosen synaptic strengths and time delays.

Real electrical synapses mediate bidirectional interactions and transfer signals only between neighboring neurons; in contrast, chemical synapses convey information unidirectionally between distantly situated neurons. To account for this, the model implements layers with bidirectional electrical coupling with nearest neighbor interactions (Figure 1A), while unidirectional chemical coupling is implemented with nonlocal interactions, i.e., also including connections other than nearest neighbor interactions (Figure 1B). These coupling topologies and interaction modes are biologically relevant and will also allow us to compare the functional role played by chemical and electrical synaptic interactions in processing information generated during SISR.

The stochastic differential equations resulting from this two-layer FHN neural network are given by

(1) $$\begin{array}{l} \begin{array}{c} \{\begin{array}{c} \text{ d}v_{l,i}=\left(v_{l,i}-\frac{v_{l,i}^{3}}{3}-w_{l,i}+E_{l,i}+M_{l,i}^{e}-C_{l,i}-M_{l,i}^{c}\right)\text{d}t\\ \;+\sigma_{l}\text{d}W_{l,i},\\ \text{d}w_{l,i}=\varepsilon \left(v_{l,i}+\alpha -\beta w_{l,i}\right)\text{d}t, \end{array} \end{array} \end{array}$$

where each neuron is represented by a node *i* = 1, …, *N* in the multiplex network with layers *l* = 1, 2, and the functional dependencies are given by,

(2) $$\begin{array}{l} \begin{array}{c} \{\begin{array}{c} \;E_{l,i}=\frac{\kappa_{l,e}}{2n_{l,e}}\overset{i+n_{l,e}}{\underset{j=i-n_{l,e}}{\sum }}\left(v_{l,j}\left(t-\tau_{l,e}\right)-v_{l,i}\left(t\right)\right),\\ \;C_{l,i}=\frac{\kappa_{l,c}}{2n_{l,c}}\left(v_{l,i}\left(t\right)-V_{\text{syn}}\right)\overset{i+n_{l,c}}{\underset{j=i-n_{l,c}}{\sum }}\\ \;{\left\{1+\exp\left[-\lambda \left(v_{l,j}\left(t-\tau_{l,c}\right)-\Theta_{\text{syn}}\right)\right]\right\}}^{-1},\\ M_{1,i}^{e}=\kappa_{m,e}\left(v_{2,i}\left(t-\tau_{m,e}\right)-v_{1,i}\left(t\right)\right),\\ M_{2,i}^{e}=\kappa_{m,e}\left(v_{1,i}\left(t-\tau_{m,e}\right)-v_{2,i}\left(t\right)\right),\\ M_{1,i}^{c}=\kappa_{m,c}\left(v_{1,i}\left(t\right)-V_{\text{syn}}\right)\\ \;{\left\{1+\exp\left[-\lambda \left(v_{2,i}\left(t-\tau_{m,c}\right)-\Theta_{\text{syn}}\right)\right]\right\}}^{-1},\\ M_{2,i}^{c}=\kappa_{m,c}\left(v_{2,i}\left(t\right)-V_{\text{syn}}\right)\\ \;{\left\{1+\exp\left[-\lambda \left(v_{1,i}\left(t-\tau_{m,c}\right)-\Theta_{\text{syn}}\right)\right]\right\}}^{-1}. \end{array} \end{array} \end{array}$$

We fixed the number of neurons per layer to *N* = 25 throughout this study. The membrane potential and the recovery current variables of neuron *i* in layer *l* are given by *v*_*l,i*_ ∈ ℝ and *w*_*l,i*_ ∈ ℝ, respectively, and 0 < ε ≪ 1 sets the timescale separation between the fast membrane potential and the slow recovery current variables. The excitability threshold β > 0 of the neurons is a codimension-one Hopf bifurcation parameter. α ∈ (0, 1) is a constant parameter. The additive noise term d*W*_*l,i*_ represents mean-centered Gaussian noise with ${\langle \text{d}W_{l,i}\left(t\right)\text{d}W_{l,i}\left(t^{\prime }\right)\rangle }_{t}=\delta \left(t-t^{\prime }\right)$ and variance (strength) σ_*l*_, and it models the synaptic fluctuations observed in neural networks.

*E*_*l,i*_ represent the electrical synaptic interactions between neurons coupled within a ring layer network with strength κ_*l,e*_ and time delay τ_*le*_, respectively, and an interaction range set to *n*_*l,e*_ = 1 since electrical synapses interact only locally. The coupling mediated by electrical synapses is of diffusive type, i.e., the electrical coupling term (intra- or inter-layer) vanishes if *v*_1,*i*_ and *v*_1,*j*_ (resp. *v*_2,*i*_ and *v*_2,*j*_) or *v*_1,*i*_ and *v*_2,*i*_ are equal.

$M_{1,i}^{e}$ and $M_{2,i}^{e}$ represent the coupling between layers via electrical synapses (i.e., electrical multiplexing of layers) with strength κ_*m,e*_ and delay τ_*m,e*_, respectively.

*C*_*l,i*_ represent chemical synaptic interactions between neurons coupled within a layer with ring topology, with strength κ_*l,c*_ and time delay τ_*l,c*_ and where 1 < *n*_*l,c*_ < (*N* − 1)/2 represents interaction range on the ring network layer; we fix *n*_*l,e*_ = 8 all through this paper. The chemical synaptic function is modeled by a sigmoidal input-output function, $\Gamma \left(v_{i}\right)=\frac{1}{1+e^{-\lambda \left(v_{i}-\Theta_{\text{syn}}\right)}}$ (see Equation 2 in Greengard, 2001), where parameter λ = 10.0 determines the slope of the function and Θ_syn_ = −0.25 the synaptic firing threshold.

$M_{1,i}^{c}$ and $M_{2,i}^{c}$ represent the coupling between layers mediated by chemical synapses (i.e., chemical multiplexing of layers) with κ_*m,c*_ and τ_*m,c*_ representing the strength and time delay, respectively. *V*_syn_ represents the synaptic reversal potential. For *V*_syn_ < *v*_*l,i*_(*t*), the chemical synaptic interaction has a depolarizing effect that makes the synapse inhibitory; for *V*_syn_ > *v*_*l,i*_(*t*), the synaptic interaction has a hyper-polarizing effect, making the synapse excitatory. For the version of the FHN neuron model used in this study, the membrane potentials |*v*_*l,i*_(*t*)| ≤ 2.0 (*l* = 1, 2; *i* = 1, 2, …, *N*) for all time *t*. For the choice of fixed *V*_syn_ = −3.0 (maintained throughout our computations), the term (*v*_*l,i*_(*t*) − *V*_syn_) in Equation (2) is always positive. So, the inhibitory and excitatory natures of chemical synapses will depend only on the sign in front of the synaptic coupling strengths κ_*l,c*_ and κ_*m,c*_. To make the chemical synapse inhibitory, we chose a negative sign i.e., when the pre-synaptic neuron spikes, it prevents the post-synaptic neuron from spiking and, conversely, a positive sign for excitatory chemical synapses.

## 3. Numerical Methods

In our numerical simulations, we used the fourth-order Runge-Kutta algorithm for stochastic processes (Kasdin, 1995) to integrate over a very long time interval (*T* = 600, 000 time units) to average time series over time with seven realizations for each noise amplitude. In the numerical simulations, this long time interval permitted us to collect with a small noise amplitude at least 125 interspike intervals with ε = 0.0005 ≪ 1. Each network layer had *N* = 25 neurons.

To measure how pronounced SISR is, we used the coefficient of variation (*R*_*T*_), which is an important statistical measure based on the time intervals between spikes. It measures the regularity of noise induced spiking and therefore a measure of how pronounced SISR can be at a particular noise amplitude. *R*_*T*_ exploits the inter-spike interval (ISI) where the *m*th interval is defined as the difference between two consecutive spike times $t_{i}^{m}$ and $t_{i}^{m+1}$ of neuron *i* in a network, namely ${\text{ISI}}_{i}=t_{i}^{m+1}-t_{i}^{m}>0$. For the *i*th neuron, the ratio between the standard deviation and the mean defines the coefficient of variation of the ISIs over a time interval [0, *T*] as (Pikovsky and Kurths, 1997):

(3) $$\begin{array}{l} R_{T_{i}}=\frac{\sqrt{\langle ISI_{i}^{2}\rangle -{\langle ISI_{i}\rangle }^{2}}}{\langle ISI_{i}\rangle }, \end{array}$$

where 〈*ISI*_*i*_〉 and $\langle ISI_{i}^{2}\rangle$ represent the mean and the mean squared inter-spike intervals of the *i*th neuron, respectively. The above definition of *R*_*T*_ is limited to characterizing SISR in an isolated neuron. For a network of coupled neurons, SISR can be measured by redefining *R*_*T*_ as follows (Masoliver et al., 2017):

(4) $$\begin{array}{l} R_{T}=\frac{\sqrt{\langle \bar{ISI^{2}}\rangle -{\langle \bar{ISI}\rangle }^{2}}}{\langle \bar{ISI}\rangle }, \end{array}$$

with

(5) $$\begin{array}{l} \begin{array}{c} \{\begin{array}{c} \langle \bar{ISI}\rangle =\frac{1}{N}\overset{N}{\underset{i=1}{\sum }}\langle ISI_{i}\rangle ,\\ \langle \bar{ISI^{2}}\rangle =\frac{1}{N}\overset{N}{\underset{i=1}{\sum }}\langle ISI_{i}^{2}\rangle , \end{array} \end{array} \end{array}$$

where the extra bar indicates the additional average over the total number of neurons *N* in the layer.

Of course, other statistical measures exist such as the correlation time, the power spectral density, and the signal-to-noise ratio which are commonly used measures to quantify the coherence of noise induced spiking activity. However, from a neurobiological point of view, *R*_*T*_ is more important than the other measures because it is related to the timing precision of the information processing in neural systems (Pei et al., 1996). Because of *R*_*T*_'s importance in neural information processing, we shall use it to characterize the regularity of the noise-induced oscillations generated by SISR in our neural network. For a Poissonian spike train (rare and incoherent spiking), *R*_*T*_ = 1. If *R*_*T*_ < 1, the sequence becomes more coherent, and *R*_*T*_ vanishes for a periodic deterministic spike train. *R*_*T*_ values greater than 1 correspond to a point process that is more variable than a Poisson process (Kurrer and Schulten, 1995; Yamakou and Jost, 2018).

## 4. Conditions for SISR in Isolated Layers in the Excitable Regime

We first consider the case of isolated layers of the multiplex networks in Figures 1A,B. Thus, neurons in such an isolated layer are connected either only via electrical synapses or via chemical synapses. In particular, here we will establish the analytic conditions necessary for the emergence of the SISR in these isolated network layers of FHN neurons in the excitable regime. From these conditions, we will furthermore obtain the minimum and maximum noise amplitudes required for SISR to occur in an isolated layer.

For SISR to occur, it is necessary to be in the excitable parameter regime. The isolated FHN neuron has a unique and stable fixed point in this regime. Choosing an initial condition in the basin of attraction of this fixed point will result in at most one large non-monotonic excursion into the phase space after which the trajectory asymptotically approaches the fixed point and stays there until initial conditions are changed again (Izhikevich, 2000; Yamakou and Jost, 2018).

Considering the multiplex networks in Figure 1 with disconnected layers (κ_*m,e*_ = κ_*m,c*_ = 0), we may place an isolated neuron (κ_*l,e*_ = 0 or κ_*l,c*_ = 0) into an excitable regime by fixing parameter α = 0.5. The bifurcation parameter β is chosen such that β > β_*h*_(ε), where β_*h*_(ε) is defined as the Hopf bifurcation value of an isolated neuron. Fixing the timescale separation parameter value to ε = 0.0005, we calculate the Hopf bifurcation value to be β_*h*_(ε) = 0.7497. It is important to note That, for β ≤ β_*h*_(ε), an isolated neuron is in the oscillatory regime—a regime that we want to avoid since the coherent oscillations generated by SISR are due only to the presence of noise rather than to the occurrence of a Hopf bifurcation (Yamakou and Jost, 2018).

Moreover, we have to ensure that the network of coupled neurons as a whole stays in the excitable regime rather than just single neurons in isolation. Indeed, certain time-delayed couplings may induce self-sustained oscillations in a network layer even though the isolated neurons remain inside the excitable regime. In layers with excitatory chemical synapses, a saddle-node bifurcation onto a limit cycle may generate self-sustained oscillations induced via time-delayed couplings (Schöll et al., 2009). On the other hand, when used for the multiplexing of layers, some values of time delays and coupling strengths of the excitatory chemical synapses cannot provoke this saddle-node bifurcation. Therefore, we did not consider excitatory chemical synapses for the coupling of neurons within layers but rather only for the coupling between layers. We therefore need to make sure that neurons connected in each network layer stay outside the parameter regime where oscillations are induced by time-delayed coupling. First, we need to determine if such a regime exists and identify it.

**Figure 2:**
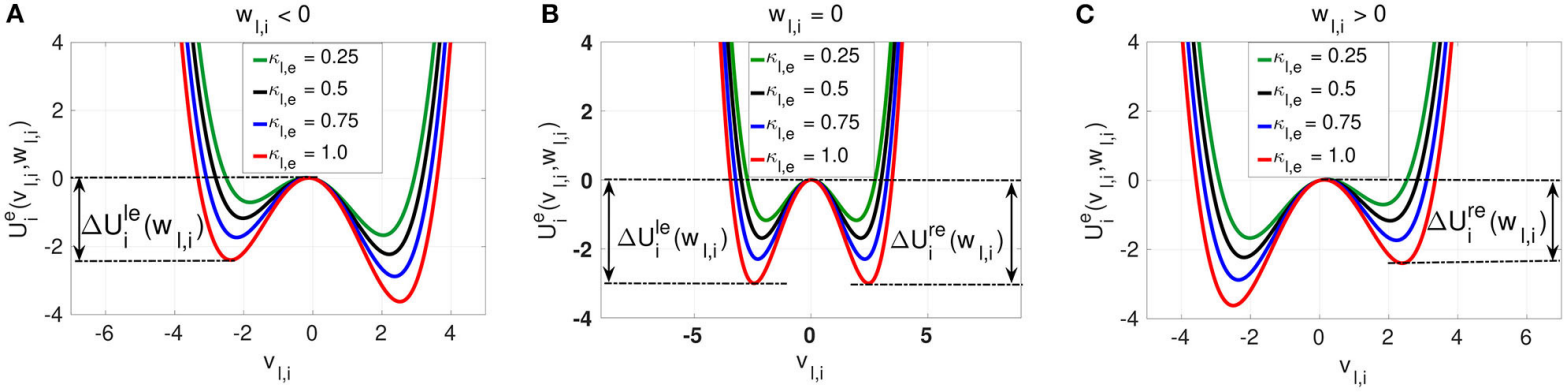
The electrical interaction potential $U_{i}^{e}\left(v_{l,i},w_{l,i}\right)$ in Equation (7) is shown for a locally coupled ring network topology (*n*_*l,e*_ = 1) with the energy barriers for the asymmetric cases (*w*_*l,i*_ ≠ 0) **(A,C)** and symmetric (*w*_*l,i*_ = 0) case **(B)**. The stronger the intra-layer synaptic strength κ_*l,e*_ is, the deeper the energy barrier functions $△U_{i}^{le}\left(w_{l,i}\right)$ and $△U_{i}^{re}\left(w_{l,i}\right)$ are. The saddle point and the left and right minima of the interaction potential are located at $v_{l,i}=v_{m}^{*}\left(w_{l,i}\right)$, $v_{l,i}=v_{l}^{*}\left(w_{l,i}\right)$, and $v_{l,i}=v_{r}^{*}\left(w_{l,i}\right)$, respectively.

**Figure 3:**
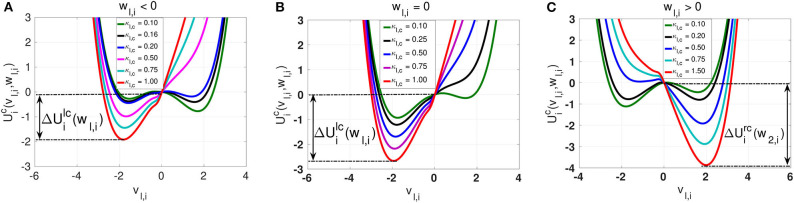
Landscapes of the inhibitory chemical interaction potential $U_{i}^{c}\left(v_{l,i},w_{l,i}\right)$ in Eq. (7) for a non-locally (*n*_*l,c*_ = 8) coupled ring network topology. The symmetry of the potential is governed not by the slow variable *w*_*l,i*_ as in the case of the electrical interaction potential, but by the chemical synaptic strength κ_*l,c*_. In **(A)** with *w*_*l,i*_ < 0, the asymmetric potential can become symmetric when the synaptic strength is κ_*l,c*_ = 0.16. In **(B)** with *w*_*l,i*_ = 0, the potential remains asymmetric for all values of the synaptic strength κ_*l,c*_. In **(C)** with *w*_*l,i*_ > 0, the asymmetric potential can again become symmetric when the synaptic strength is κ_*l,c*_ = 0.20. Similarly to the electrical synaptic strength, the stronger the intra-layer chemical synaptic strength κ_*l,c*_ is, the deeper the energy barrier functions $△U_{i}^{lc}\left(w_{l,i}\right)$ and $△U_{i}^{rc}\left(w_{l,i}\right)$ are.

Taking the limit ε → 0 in the isolated layer *l* = 1, 2 (κ_*m,e*_ = κ_*m,c*_ = 0) for either electrical (κ_*l,c*_ = 0) or chemical synapses (κ_*l,e*_ = 0) only, the equations for each neuron in this layer reduces to coupled Langevin equations of the form,

(6) $$\begin{array}{l} \text{d}v_{l,i}=-\frac{\partial U_{i}^{e,c}\left(v_{l,i},w_{l,i}\right)}{\partial v_{l,i}}\text{d}t+\sigma_{l}\text{d}W_{l,i}, \end{array}$$

where the electrical $U_{i}^{e}\left(v_{l,i},w_{l,i}\right)$ and chemical $U_{i}^{c}\left(v_{l,i},w_{l,i}\right)$ interaction potentials (*i* = 1, …, *N*) are double-well potentials given by Equation (7) and may be viewed as functions of *v*_*l,i*_ where *w*_*l,i*_ is nearly constant. Figures 2, 3, respectively show the modulation of landscapes of electrical and chemical interaction potentials with changing synaptic strength.

(7) $$\begin{array}{l} \left\{ \begin{array}{l} U_{i}^{e}(v_{l,i},w_{l,i})=\frac{1}{12}v_{l,i}^{4}-\frac{1}{2}v_{l,i}^{2}+v_{l,i}w_{l,i}-\frac{\kappa_{l,e}}{2n_{l,e}}\\ \;\sum_{j=i-n_{l,e}}^{i+n_{l,e}}\left(v_{l,i}(t)v_{l,j}(t-\tau_{l,e})-\frac{1}{2}v_{l,i}{\left(t\right)}^{2}\right),\\ U_{i}^{c}(v_{l,i},w_{l,i})=\frac{1}{12}v_{l,i}^{4}-\frac{1}{2}v_{l,i}^{2}+v_{l,i}w_{l,i}\\ \;+\frac{\kappa_{l,c}}{2n_{l,c}}\sum_{j=i-n_{l,c}}^{i+n_{l,c}}\frac{1}{2}v_{l,i}(t)(v_{l,i}(t)-2V_{\text{syn}})\\ \;{\left\{1+\exp[-\lambda (v_{l,j}(t-\tau_{l,c})-\Theta_{\text{syn}})]\right\}}^{-1}, \end{array}\right. \end{array}$$

We observe three different behaviors for the electrical potential interaction $U_{i}^{e}\left(v_{l,i},w_{l,i}\right)$. (i) When *w*_*l,i*_ < 0, we find that $U_{i}^{e}\left(v_{l,i},w_{l,i}\right)$ is asymmetric with the shallower well on the left. The neuron is close to the stable homogeneous fixed point at $\left(v_{l,i}^{*},w_{l,i}^{*}\right)=\left(-1.003975,-0.666651\right)$, and a spike consists of jumping over the left energy barrier $△U^{le}\left(w_{l,i}\right)$ into the right well (see Figure 2A). (ii) When *w*_*l,i*_ = 0, then $U_{i}^{e}\left(v_{l,i},w_{l,i}\right)$ is symmetric with $△U^{le}\left(w_{l,i}\right)=△U^{re}\left(w_{l,i}\right)$, and the neuron is half way between the quiescent state and the spike state (see Figure 2B). (iii) When *w*_*l,i*_ > 0, then $U_{i}^{e}\left(v_{l,i},w_{l,i}\right)$ is also asymmetric. The neuron has spiked and a return to the quiescent state (the homogeneous fixed point) consists of jumping over the right energy barrier $△U^{re}\left(w_{l,i}\right)$ into the left well (see Figure 2C). The intra-layer electrical synapse κ_*l,e*_ does not change the symmetry (or asymmetry) of the interaction potential $U_{i}^{e}\left(v_{l,i},w_{l,i}\right)$. It only changes the depth of the energy barriers. The stronger κ_*l,e*_ is, the deeper the energy barrier functions $△U^{le}\left(w_{l,i}\right)$ and $△U^{re}\left(w_{l,i}\right)$ defined in Equation (9) are.

The chemical potential interaction $U_{i}^{c}\left(v_{l,i},w_{l,i}\right)$ shows richer landscape dynamics due to its stronger nonlinearity. We first notice that, just like with intra-layer electrical synaptic strength κ_*l,e*_, intra-layer inhibitory chemical synaptic strength κ_*l,c*_ changes the depth of the energy barriers $△U_{i}^{lc}\left(w_{l,i}\right)$ and $△U_{i}^{rc}\left(w_{l,i}\right)$. That is, the stronger κ_*l,c*_ is, the deeper the energy barriers $△U_{i}^{lc}\left(w_{l,i}\right)$ and $△U_{i}^{rc}\left(w_{l,i}\right)$ are. In contrast to the electrical synaptic strength κ_*l,e*_, the inhibitory chemical synaptic strength κ_*l,c*_ is capable of changing the symmetry or (asymmetry) of the chemical potential $U_{i}^{c}\left(v_{l,i},w_{l,i}\right)$, where we distinguish the following cases: (i) When *w*_*l,i*_ < 0, then $U_{i}^{c}\left(v_{l,i},w_{l,i}\right)$ can be symmetric or asymmetric depending on the value of the inhibitory chemical synaptic strength κ_*l,c*_. If *w*_*l,i*_ < 0 and κ_*l,c*_ = 0.16, we see from Figure 3A that $U_{i}^{c}\left(v_{l,i},w_{l,i}\right)$ is symmetric and becomes asymmetric as κ_*l,c*_ changes. (ii) When *w*_*l,i*_ = 0.0, we do not have any symmetric chemical potential landscape as shown in Figure 3B, contrasting our observations for the electrical potential. (iii) For *w*_*l,i*_ > 0 (see Figure 3C), the chemical potential landscape is symmetric for κ_*l,c*_ = 0.2 and becomes asymmetric as κ_*l,c*_ changes. Moreover, we notice that, for values of the chemical synaptic strength κ_*l,c*_ for which the chemical interaction potential is symmetric, the energy barriers functions are shallower than in the symmetric case of the electrical potential. The important common feature of the electrical and inhibitory chemical potential is the deepening of the energy barriers $△U_{i}^{le,c}\left(w_{l,i}\right)$ and $△U_{i}^{re,c}\left(w_{l,i}\right)$ with increase in the intra-layer electrical κ_*l,e*_, and inhibitory chemical κ_*l,c*_ synaptic strengths shall explain why SISR is deteriorated by stronger intra-layer synaptic connections.

We choose parameters of the coupled neurons in Equation (6) such that they satisfy the conditions necessary for the occurrence of SISR. These conditions are adapted from those valid for an isolated FHN neuron (DeVille et al., 2005; Yamakou and Jost, 2018) so that they include (one at a time) the time-delayed electrical and inhibitory chemical synaptic connections between the FHN neurons coupled in a ring network. The resulting conditions are

(8) $$\begin{array}{l} \{\begin{array}{l} \underset{\left(\varepsilon ,\sigma_{l})\to (0,0\right)}{\lim}\frac{\sigma_{l}^{2}}{2}\ln(\varepsilon^{-1})\in \left(△U_{i}^{le}(w_{l,i}^{*}),F_{e}(\kappa_{l,e},\tau_{l,e},n_{l,e})\right),\\ \underset{\left(\varepsilon ,\sigma_{l})\to (0,0\right)}{\lim}\frac{\sigma_{l}^{2}}{2}\ln(\varepsilon^{-1})\in \left(△U_{i}^{lc}(w_{l,i}^{*}),F_{c}(\kappa_{l,c},\tau_{l,c},n_{l,c})\right),\\ \underset{\left(\varepsilon ,\sigma_{l})\to (0,0\right)}{\lim}\frac{\sigma_{l}^{2}}{2}\ln(\varepsilon^{-1})=O(1),\\ \beta -\beta_{h}(\varepsilon )>0, \end{array} \end{array}$$

where

(9) $$\begin{array}{l} \{\begin{array}{l} F_{e}(\kappa_{l,e},\tau_{l,e},n_{l,e}):\;=\{(\kappa_{l,e},\tau_{l,e},n_{l,e}):△U_{i}^{le}(w_{l,i})=△U_{i}^{re}(w_{l,i})\},\\ \begin{array}{l} F_{c}(\kappa_{l,c},\tau_{l,c},n_{l,c}):\;=\{(\kappa_{l,c},\tau_{l,c},n_{l,c}):△U_{i}^{lc}(w_{l,i})\text{ or }△U_{i}^{rc}(w_{l,i})\\ \text{ is maximum}\}, \end{array}\\ △U_{i}^{le,c}(w_{l,i}):\;=U_{i}^{e,c}(v_{m}^{*}(w_{l,i}),w_{l,i})-U_{i}^{e,c}(v_{l}^{*}(w_{l,i}),w_{l,i}),\\ △U_{i}^{re,c}(w_{l,i}):\;=U_{i}^{e,c}(v_{m}^{*}(w_{l,i}),w_{l,i})-U_{i}^{e,c}(v_{r}^{*}(w_{l,i}),w_{l,i}), \end{array} \end{array}$$

with

(10) $$\begin{array}{l} v_{l,m,r}^{*}(w_{l,i}):=\;\{v_{l,i}:v_{l,i}-\frac{v_{l,i}^{3}}{3}-w_{l,i}+\frac{\kappa_{l,e}}{2n_{l,e}}\\ \;\sum_{j=i-n_{l,e}}^{i+n_{l,e}}\left(v_{l,j}(t-\tau_{l,e})-v_{l,i}(t)\right)=0\}, \end{array}$$

for electrical synapses and

(11) $$\begin{array}{l} v_{l,m,r}^{*}(w_{l,i}):=\{v_{l,i}:v_{l,i}-\frac{v_{l,i}^{3}}{3}-w_{l,i}-\frac{\kappa_{l,c}}{2n_{l,c}}\sum_{j=i-n_{l,c}}^{i+n_{l,c}}\left(v_{l,i}-V_{\text{syn}}\right)\\ {\left\{1+\exp[-\lambda (v_{l,j}(t-\tau_{m,c})-\Theta_{\text{syn}})]\right\}}^{-1}=0\}, \end{array}$$

for chemical synapses. Furthermore, the solution sets of Equations (10) and (11) are such that $v_{l}^{*}\left(w_{l,i}\right)<v_{m}^{*}\left(w_{l,i}\right)<v_{r}^{*}\left(w_{l,i}\right)$ define the left stable, middle unstable, and right stable branches of the cubic nullcline of each FHN neuron.

The energy barrier functions $△U_{i}^{le,c}\left(w_{l,i}\right)$ and $△U_{i}^{re,c}\left(w_{l,i}\right)$ can be obtained from the electrical interaction potential $U_{i}^{e}\left(v_{l,i},w_{l,i}\right)$ and the inhibitory chemical interaction potential $U_{i}^{c}\left(v_{l,i},w_{l,i}\right)$ by taking the difference between the potential function value at the saddle point $v_{m}^{*}\left(w_{l,i}\right)$ and at the local minima $v_{l,r}^{*}\left(w_{l,i}\right)$ of these interaction potentials (Yamakou and Jost, 2018). The energy barriers $△U_{i}^{le}(w_{l,i}^{*})$ or $△U_{i}^{lc}(w_{l,i}^{*})$ (which has to be crossed to induce a spike) is the value of the left energy barrier function at the *w*_*l,i*_-coordinate of the stable homogeneous steady state $\left[v_{l,i}^{*}\left(\kappa_{l,e},\tau_{l,e},n_{l,e}\right),w_{l,i}^{*}\left(\kappa_{l,e},\tau_{l,e},n_{l,e}\right)\right]$ or $\left[v_{l,i}^{*}\left(\kappa_{l,c},\tau_{l,c},n_{l,c}\right),w_{l,i}^{*}\left(\kappa_{l,c},\tau_{l,c},n_{l,c}\right)\right]$, respectively. This is where the electrical $△U_{i}^{le}[w_{l,i}^{*}\left(\kappa_{l,e},\tau_{l,e},n_{l,e}\right)]$ and chemical $△U_{i}^{lc}[w_{l,i}^{*}\left(\kappa_{l,c},\tau_{l,c},n_{l,c}\right)]$ energy barrier functions get their κ_*l,e*_, τ_*l,e*_, *n*_*l,e*_ and κ_*l,c*_, τ_*l,c*_, *n*_*l,c*_ dependence from.

Now from the first two conditions of Equation (8), we obtain the noise amplitude range $\left[\sigma_{l}^{\text{min}},\sigma_{l}^{\text{max}}\right]$ within which SISR occurs in the layer network of electrically (chemically) coupled FHN neurons:

(12) $$\begin{array}{l} \{\begin{array}{l} \sigma_{l}^{{\text{min}}^{e}}=\sqrt{\frac{2△U_{i}^{le}(w_{l,i}^{*}(\kappa_{l,e},\tau_{l,e},n_{l,e}))}{\ln(\varepsilon^{-1})}},\\ \sigma_{l}^{{\text{max}}^{e}}=\sqrt{\frac{2F_{e}(\kappa_{l,e},\tau_{l,e},n_{l,e})}{\ln(\varepsilon^{-1})}}.\\ \sigma_{l}^{{\text{min}}^{c}}=\sqrt{\frac{2△U_{i}^{lc}(w_{l,i}^{*}(\kappa_{l,c},\tau_{l,c},n_{l,c}))}{\ln(\varepsilon^{-1})}},\\ \sigma_{l}^{{\text{max}}^{c}}=\sqrt{\frac{2F_{c}(\kappa_{l,c},\tau_{l,c},n_{l,c})}{\ln(\varepsilon^{-1})}}. \end{array} \end{array}$$

We observe that $\sigma_{l}^{{\text{min}}^{e}}$ and $\sigma_{l}^{{\text{min}}^{c}}$ depend on the fixed point coordinate $w_{l,i}^{*}\left(\kappa_{l,e},\tau_{l,e},n_{l,e}\right)$ and $w_{l,i}^{*}\left(\kappa_{l,c},\tau_{l,c},n_{l,c}\right)$ which in turn also depends on the synaptic parameters κ_*l,e*_, τ_*l,e*_, *n*_*l,e*_ and κ_*l,c*_, τ_*l,c*_, *n*_*l,c*_, respectively. Therefore, changing (κ_*l,e*_, τ_*l,e*_, *n*_*l,e*_) or (κ_*l,c*_, τ_*l,c*_, *n*_*l,c*_) will change the value of $w_{l,i}^{*}\left(\kappa_{l,e},\tau_{l,e},n_{l,e}\right)$ or $w_{l,i}^{*}\left(\kappa_{l,c},\tau_{l,c},n_{l,c}\right)$, which will in turn change the value of $\sigma_{l}^{{\text{min}}^{e}}$ or $\sigma_{l}^{{\text{min}}^{c}}$ via the energy barrier function $△U_{i}^{le}\left(w_{l,i}^{*}\right)$ or $△U_{i}^{lc}\left(w_{l,i}^{*}\right)$, respectively. However, because of the local nature electrical synapses and non-locality of the chemical synapses, we fixed *n*_*l,e*_ = 1 and *n*_*l,c*_ = 8 throughout our numerical computations. Hence, the two control parameters used are the synaptic time-delayed couplings (τ_*l,e*_, κ_*l,e*_) and (τ_*l,c*_, κ_*l,c*_). On the other boundary, $\sigma_{l}^{{\text{max}}^{e}}$ and $\sigma_{l}^{{\text{max}}^{c}}$ do not depend on the coordinates of the stable homogeneous fixed point but on the complicated functions *F*_*e*_(κ_*l,e*_, τ_*l,e*_, *n*_*l,e*_) and *F*_*c*_(κ_*l,c*_, τ_*l,c*_, *n*_*l,c*_), completely defined in Equations (9), (10), and (11). Knowing the minimum and maximum range of the noise amplitude within which SISR occurs will be very useful in discussing the numerical results in the following sections.

## 5. SISR in Isolated Layers

### 5.1. SISR in Isolated Layers With Electrical Synapses Only

**Figure 4:**
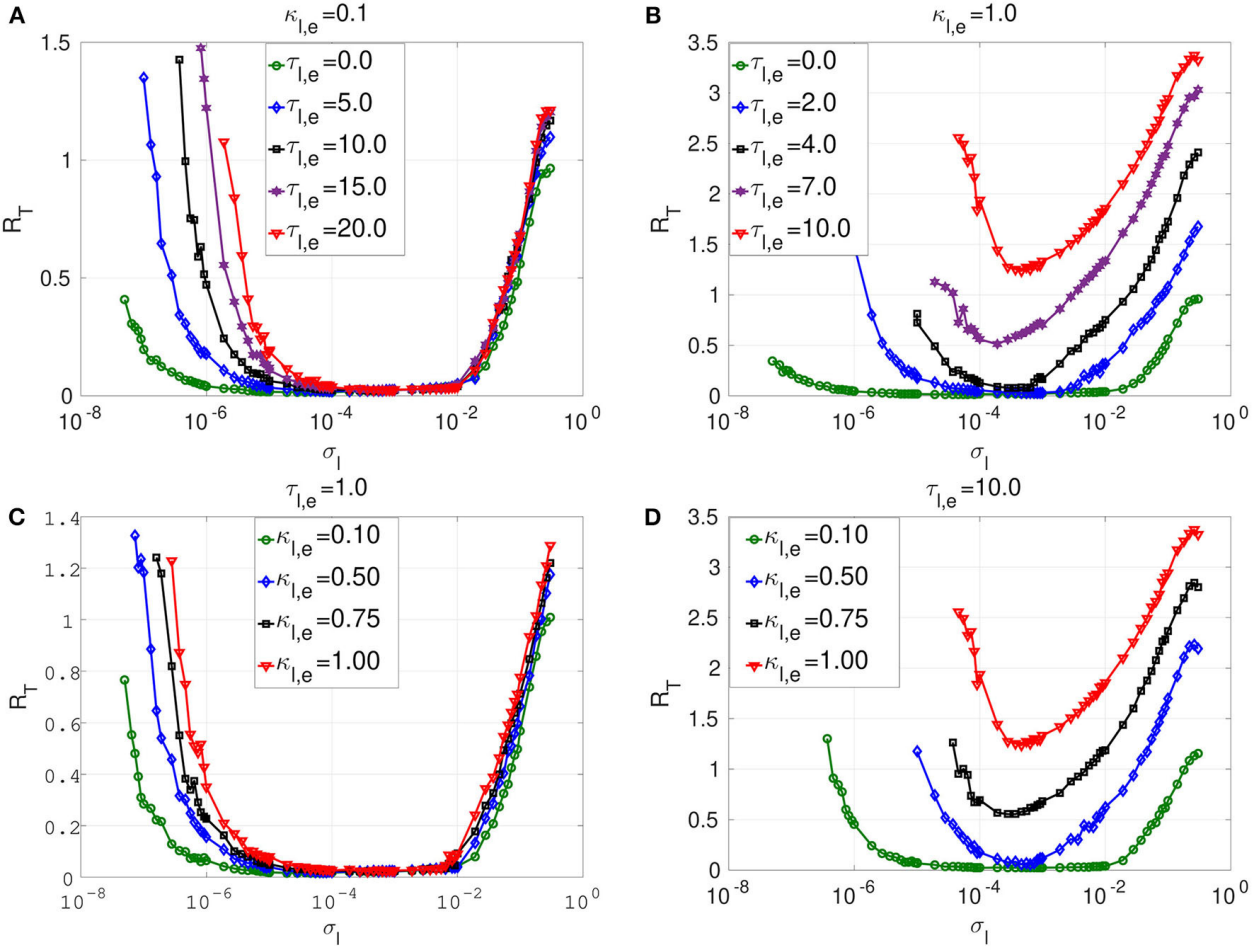
Coefficient of variation *R*_*T*_ against noise amplitude σ_*l*_ of layer *l* in isolation. In **(A)** and **(B)**, we have the *R*_*T*_ curves of weak and strong electrical synaptic strengths κ_*l,e*_, respectively, for short, intermediate and long synaptic time delays τ_*l,e*_. In **(C)** and **(D)**, we have the *R*_*T*_ curves of short and relatively long synaptic time delays τ_*l,e*_, respectively, for weak, intermediate and strong synaptic strengths κ_*l,e*_. Increasing (decreasing) the electrical synaptic strength κ_*l,e*_ or the length of its time delay τ_*l,e*_, deteriorates (enhances) SISR by increasing (decreasing) the values of *R*_*T*_ and by shrinking (extending) the interval of the noise amplitude in which *R*_*T*_ can achieve very low values. For example, in **(D)**, for κ_*l,e*_ = 1.0 and τ_*l,e*_ = 10.0, the red *R*_*T*_-curve lies entirely above the line *R*_*T*_ = 1.0 with a lowest value of *R*_*T*_min__ = 1.24 occurring at just one point $\sigma_{l}=4.6\times 10^{-4}$, indicating the non-existence of SISR. Parameters of layer *l*: *N* = 25, *n*_*l,e*_ = 1, β = 0.75, ε = 0.0005, α = 0.5.

We begin our numerical study with the dynamics of layer *l* in isolation, where neurons are connected only via local electrical synapses in a ring network topology, i.e., we consider Equation (1) with *n*_*l,e*_ = 1, κ_*l,e*_ ≠ 0 and κ_*m,e*_ = κ_*m,c*_ = κ_*l,c*_ = 0. Figure 4 shows the variation of *R*_*T*_ against the noise amplitude σ_*l*_ for this layer. In the numerical computations, we choose ε = 0.0005 ≪ 1 because SISR can only occur in the singular limit, ε → 0, and the weak noise limit, σ_*l*_ → 0, imposed by Equation (8).

In Figure 4A, a weak electrical synaptic strength is considered fixed, κ_*l,e*_ = 0.1. All the flat-bottom *R*_*T*_-curves obtained with different time delays (τ_*l,e*_ = 0.0, τ_*l,e*_ = 5.0, τ_*l,e*_ = 10.0, τ_*l,e*_ = 15.0, τ_*l,e*_ = 20.0) show a deep and broad minimum, indicating that the spike train has a high degree of coherence due to SISR for a wide range of the noise amplitude. We notice that even though the minimum (and low) values of *R*_*T*_ stays constant for various time delays, the left branch of the *R*_*T*_-curve is significantly being shifted to the right as the time delay increases. This means that with weak electrical synapses, the coherence of the spiking activity due to SISR is not affected as the time delay becomes longer, but the coherence is achieved only at relatively larger noise amplitudes σ_*l*_. Thus, we can obtain the same degree of SISR with longer time delays provided we increase the noise amplitude (within the interval given in Equation 12) as the time delay increases. In Figure 4A, we have approximately the same minimum value of *R*_*T*_min__ ≈ 0.015 for: τ_*l,e*_ = 0.0 with $\sigma_{l}\in (3.7\times 10^{-7},1.9\times 10^{-2})$; τ_*l,e*_ = 5.0 with $\sigma_{l}\in (2.8\times 10^{-6},1.9\times 10^{-2})$; τ_*l,e*_ = 10.0 with $\sigma_{l}\in (5.5\times 10^{-6},1.0\times 10^{-2})$; τ_*l,e*_ = 15.0 with $\sigma_{l}\in (1.9\times 10^{-5},1.0\times 10^{-2})$; and τ_*l,e*_ = 20.0 with $\sigma_{l}\in (2.8\times 10^{-5},1.0\times 10^{-2})$. We note that the lower bound of the noise intervals increases as the time delay increases while the upper bounds are almost fixed.

In Figure 4B, we consider a strong electrical synapse (κ_*l,e*_ = 1.0). We observe that, in contrast to Figure 4A with a weak electrical synapse, increasing the time delay squeezes the left and right branches of the *R*_*T*_-curves into a smaller noise interval, while shifting the curves to higher values, thus deteriorating SISR. In Figure 4B, we have different noise intervals for different minima of *R*_*T*_: *R*_*T*_min__ = 0.015 at τ_*l,e*_ = 0.0 for $\sigma_{l}\in (2.8\times 10^{-7},2.9\times 10^{-2})$; *R*_*T*_min__ = 0.029 at τ_*l,e*_ = 2.0 for $\sigma_{l}\in (2.8\times 10^{-5},2.8\times 10^{-3})$; *R*_*T*_min__ = 0.078 at τ_*l,e*_ = 4.0 for $\sigma_{l}\in (1.9\times 10^{-4},6.4\times 10^{-4})$. In the last two cases, the noise intervals, in which we have the most deteriorated SISR, have shrunk to points with *R*_*T*_min__ = 0.51 at $\sigma_{l}=1.9\times 10^{-4}$ for τ_*l,e*_ = 7.0; and *R*_*T*_min__ = 1.24 at $\sigma_{l}=4.6\times 10^{-4}$ for τ_*l,e*_ = 10.0. We thus see that, with strong electrical synapses, the effect of the time delay on SISR becomes significant, unlike when the electrical synapse is weak as in Figure 4A. In Figure 4B, we observe that even though the *R*_*T*_-curves for τ_*l,e*_ = 7.0 and τ_*l,e*_ = 10.0 are non-monotonic (characteristic of the existence of an optimal noise value for coherence), the minimum values of these curves are high (0.51 and 1.24, respectively). Here, at only τ_*l,e*_ = 10.0, *R*_*T*_min__ is already above 1.0 (indicating a stochastic spiking activity that is more variable than the Poisson process), whereas with weak electrical synapses in Figure 4A, even at τ_*l,e*_ = 20.0, we still have *R*_*T*_min__ ≈ 0.015.

In Figures 4C,D, we vary the electrical synaptic strength while the synaptic time is fixed at a short (τ_*l,e*_ = 1.0) and a long (τ_*l,e*_ = 10.0) delay, respectively. A similar behavior as in Figures 4A,B is observed, with weak and strong electrical synaptic strengths, respectively. That is, at short synaptic time delays (see Figure 4C), the *R*_*T*_-curves show a deep and broad minimum, indicating a high degree of coherence due to SISR for a wide range of the noise amplitude when the electrical synaptic strength κ_*l,e*_ is varied. Here, as κ_*l,e*_ increases, and only the left branches of the *R*_*T*_-curves are shifted to the right, while the right branch of the *R*_*T*_-curves are fixed, thereby fixing the upper bound of the noise amplitude σ_*l*_ below which SISR is optimal. This means that at short electrical time delays, the coherence of the spiking activity due to SISR is not affected as the electrical synaptic strength becomes stronger, but the coherence is achieved only at relatively larger noise amplitudes σ_*l*_. In Figure 4D, where electrical synaptic time delays are longer, increasing the electrical synaptic strength not only increases the minimum value of the *R*_*T*_-curves (thereby deteriorating SISR), but also shrinks the size of the noise interval in which SISR is optimized on both ends.

The response of SISR to changes in the synaptic strength κ_*l,e*_ and time delay τ_*l,e*_ in Figure 4 can be explained in terms of the electrical interaction potential $U_{i}^{e}\left(v_{l,i},w_{l,i}\right)$ given in Equation (7) and represented in Figure 2. We observe in Figure 2 that, for a fixed (*n*_*l,e*_ = 1) ring network topology and time delay τ_*l,e*_, as the synaptic strength κ_*l,e*_ increases, the energy barriers $△U_{i}^{le}\left(w_{l,i}\right)$ and $△U_{i}^{re}\left(w_{l,i}\right)$ become deeper. In particular, when *w*_*l,i*_ < 0, the trajectory is in the left potential well and as κ_*l,e*_ becomes stronger (0.25, 0.5, 0.75, 1.0), the left energy barrier $△U_{i}^{le}\left(w_{l,i}\right)$ becomes deeper (hence the trajectory at the bottom of the well get closer to the homogeneous stable fixed point at $w_{l,i}^{*}=-0.666651$). The deeper the left energy barrier $△U_{i}^{le}\left(w_{l,i}\right)$ is (in other words, the stronger the electrical synaptic strength κ_*l,e*_ is), therefore, the closer the trajectory to the stable fixed point is and the further away the neural system from the oscillatory regime is. For the trajectory to jump over a high energy barrier $△U_{i}^{le}\left(w_{l,i}\right)$, a stronger noise amplitude σ_*l*_ is of course needed. This is why in Figure 4 as κ_*l,e*_ increases, the left branch of the *R*_*T*_-curve is shifted to the right, meaning that stronger noise amplitudes are required to induce frequent spiking (i.e., frequent escaping from the deep left energy barrier). But, as the noise amplitude becomes bigger, the condition in Equation (8) requiring σ_*l*_ → 0 for the occurrence of SISR is violated. Hence, SISR disappears with increasing synaptic strength.

We can also see from Figure 4D that at longer time delay τ_*l,e*_, this effect (the shifting of the left branch of the *R*_*T*_-curve to the right) is more pronounced than in Figure 4C with a shorter time delay. This is because, in Equation (7), the longer the time delay is (τ_*l,e*_ ≫ 0), the further away is the quantity $\left[v_{l,i}\left(t\right)v_{l,j}\left(t-\tau_{l,e}\right)-v_{l,i}{\left(t\right)}^{2}\right]$ from zero (since neurons are identical); hence, the stronger is the effect of the synaptic strength κ_*l,e*_ on the electrical interaction potential, the energy barrier functions, and, consequently, on the *R*_*T*_-curves. Otherwise, if τ_*l,e*_ → 0, then because the neurons are identical, $[v_{l,i}\left(t\right)v_{l,j}\left(t-\tau_{l,e}\right)-v_{l,i}{\left(t\right)}^{2}]\to 0$, and κ_*l,e*_ will have little effect on the electrical interaction potential, the energy barriers functions, and consequently on the *R*_*T*_-curves. This is why the synaptic strength κ_*l,e*_ has a stronger effect on SISR only when τ_*l,e*_ gets longer, and vice versa. This theoretical explanation will also support the behavior of the time-delayed chemical synapses in the optimization of SISR as we shall see further below.

Secondly, at weak electrical synaptic strengths and short time delays (Figures 4A,C), the upper bound of the noise interval for which the *R*_*T*_-curves achieve their minima is almost constant. Here, only the lower bound of the noise intervals is shifted to the right. Whereas, at strong electrical synaptic strengths and long time delays (Figures 4B,D), both the lower and upper bounds of the noise intervals are shifted to the right and to t4he left as τ_*l,e*_ and κ_*l,e*_ increase, respectively. This has the overall effect of shrinking the noise interval in which the *R*_*T*_-curves achieve their minima to a single value of σ_*l*_. This behavior can be explained in terms of the minimum and maximum noise amplitudes between which SISR occurs obtained in Equation (12).

We observe from Equation (12) that $\sigma_{l}^{{\text{min}}^{e}}$ depends on the fixed point coordinate $w_{l,i}^{*}\left(\kappa_{l,e},\tau_{l,e},n_{l,e}\right)$, which, in turn, also depends on κ_*l,e*_, τ_*l,e*_, and *n*_*l,e*_ = 1. Therefore, changing κ_*l,e*_ and τ_*l,e*_ will change the value of $w_{l,i}^{*}\left(\kappa_{l,e},\tau_{l,e},n_{l,e}\right)$, which will, in turn, change the value of $\sigma_{l}^{{\text{min}}^{e}}$ via the energy barrier function $△U_{i}^{le}\left(w_{l,i}^{*}\right)$. Numerical computations indicate that $\sigma_{l}^{{\text{min}}^{e}}$ increases as κ_*l,e*_ and τ_*l,e*_ increase (see Figure 4). On the other boundary, $\sigma_{l}^{{\text{max}}^{e}}$ does not depend on the coordinates of the homogeneous stable fixed point, but on the complicated function *F*_*e*_(κ_*l,e*_, τ_*l,e*_, *n*_2,*e*_), fully determined by Equations (9) and (10). In Figures 4A,C (i.e., in the regimes of weak electrical synaptic strength and short time delays, respectively), we notice that $\sigma_{l}^{{\text{max}}^{e}}\approx 10^{-2}$ is nearly constant for all values of the time delay and electrical synaptic strength used. In Yamakou and Jost (2018), where a single isolate FHN neuron is considered, such fixation of the upper bound of the noise interval in which SISR occurs was already observed. In the case of a single isolated FHN neuron, the function *F*_*e*_ in Equation (9) takes a simple constant value $F_{e}=\frac{3}{4}$. This implies (for a fixed ε = 0.0005) a fixed value for $\sigma_{l}^{{\text{max}}^{e}}=[3/2\cdot \log_{e}\left(\varepsilon^{-1}\right){]}^{1/2}$.

In the case where a network of coupled FHN neurons is considered, the fixation of the upper bound of the noise interval for which SISR occurs can only be observed if *F*_*e*_(κ_*l,e*_, τ_*l,e*_, *n*_2,*e*_) → *C*, where *C* is a constant. In particular, in a weak electrical synaptic regime (κ_*l,e*_ → 0) and short time delay (τ_*l,e*_ → 0) regime (or more precisely, $\left[v_{l,i}v_{l,j}\left(t-\tau_{l,e}\right)-v_{l,i}^{2}\left(t\right)\right]\to 0$ as τ_*l,e*_ → 0, because all the neurons are identical), $F\left(\kappa_{l,e},\tau_{l,e},n_{l,e}\right)\to \frac{3}{4}$. In these regimes (see Figures 4A,C), we observe that $\sigma_{l}^{{\text{max}}^{e}}\approx 10^{-2}$, corresponding to the value obtained in Yamakou and Jost (2018) for the case of a single isolated FHN neuron (κ_*l,e*_ = 0). In the regimes of strong coupling (κ_*l,e*_ ≫ 0) and of long time delays ($\tau_{l,e}\gg 0\Rightarrow \left[v_{l,i}v_{l,j}\left(t-\tau_{l,e}\right)-v_{l,i}^{2}\left(t\right)\right]\neq 0$) shown in Figures 4B,D, the function *F*_*e*_ in Equation (9) is now strongly modified by the large values of κ_*l,e*_ and τ_*l,e*_. This is why in these regimes, the upper bound $\sigma_{l}^{{\text{max}}^{e}}$ of the noise interval, for which SISR occurs, is not fixed any longer but shifted to the left as τ_*l,e*_ and κ_*l,e*_ take on larger values. In the case of chemical synapses, as we shall see later, the same theoretical explanation holds for the shrinking, on both ends, of the interval of the noise amplitude in which SISR is optimized. Later, we shall focus on layer *l* = 2 with a non-existent SISR when it is in isolation (κ_2,*e*_ = 1.0 and τ_2,*e*_ = 10.0; see the red curve in Figure 4D with *R*_*T*_min__ > 1) and then investigate which multiplexing configuration can best optimize SISR in this layer when it is multiplexed with layer *l* = 1 when it already exhibits pronounced SISR.

### 5.2. SISR in Isolated Layers With Inhibitory Chemical Synapses Only

**Figure 5:**
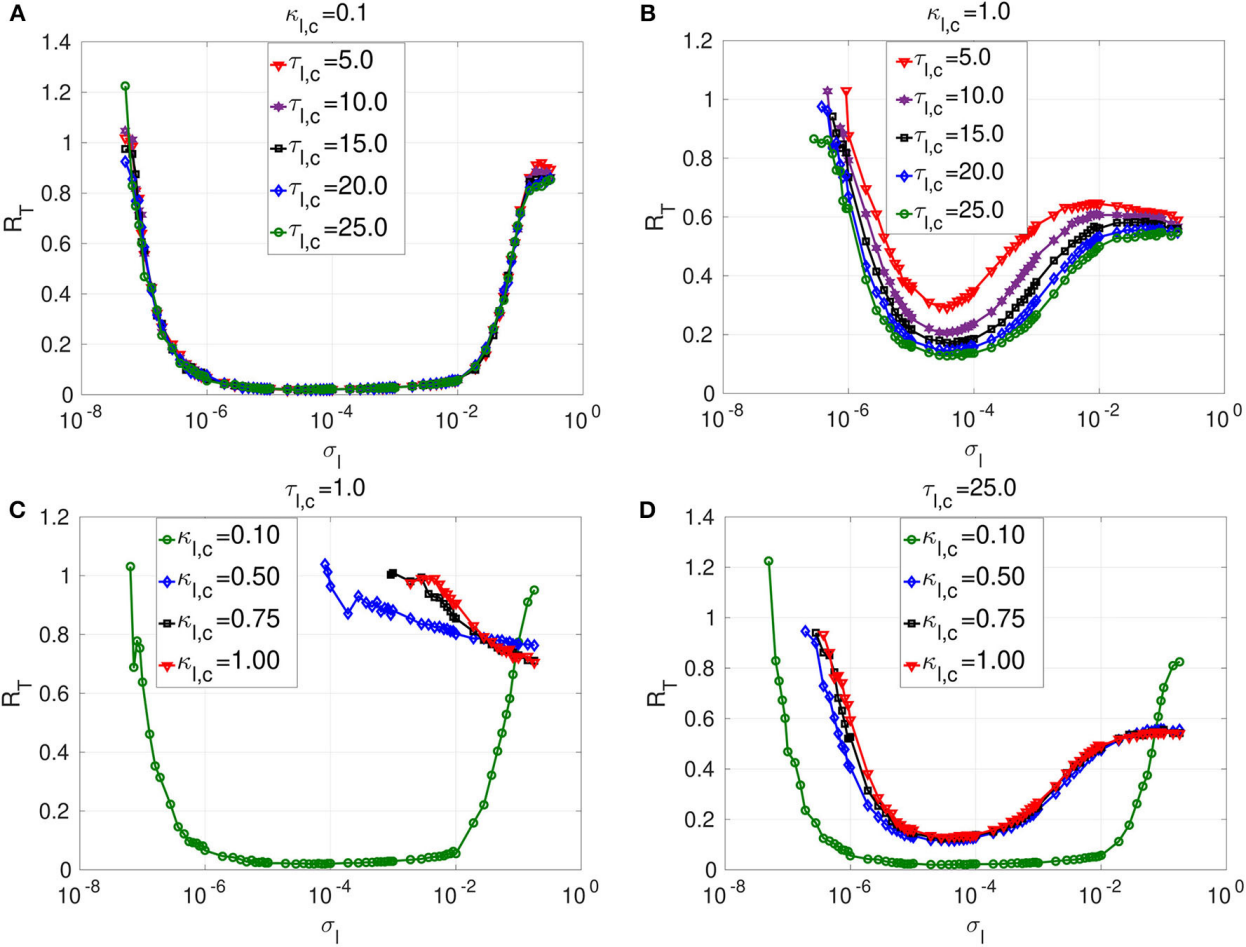
Coefficient of variation *R*_*T*_ vs noise amplitude σ_*l*_ of layer *l* in isolation. In **(A)** and **(B)**, we have the *R*_*T*_ curves of weak and strong chemical synaptic strengths κ_*l,c*_, respectively, for short, intermediate and long synaptic time delays τ_*l,c*_. In **(C)** and **(D)**, we have the *R*_*T*_ curves of short and relatively long synaptic time delays τ_*l,c*_, respectively, for weak, intermediate and strong synaptic strengths κ_*l,c*_. Increasing (decreasing) the inhibitory chemical synaptic strength κ_*l,c*_ deteriorates (enhances) SISR by increasing (decreasing) the values of *R*_*T*_ and by shrinking (extending) the interval of the noise amplitude in which *R*_*T*_ can achieve very low values. Thus, inhibitory chemical synaptic strength qualitatively behaves as the electrical synaptic strength in optimizing SISR. However, electrical synaptic and inhibitory chemical synaptic time delays show opposite behaviors in the enhancement of SISR. Decreasing (increasing) the length of inhibitory chemical time delays τ_*l,c*_, deteriorates (enhances) SISR by increasing (decreasing) the values of *R*_*T*_ and by shrinking (extending) the interval of the noise amplitude in which *R*_*T*_ can achieve very low values. This effect is particularly pronounced when the chemical synaptic strength is strong. For example, in **(C)**, for κ_*l,c*_ = 1.0 and τ_*l,e*_ = 1.0, the red *R*_*T*_-curve achieves relatively high minimum value *R*_*T*_min__ = 0.71 occurring at a relatively large noise amplitude $\sigma_{l}=4.6\times 10^{-4}$, indicating a very poor SISR. Parameters for layer *l* are: *N* = 25, *n*_*l,c*_ = 8, β = 0.75, ε = 0.0005, α = 0.5.

We investigated the dynamics of layer *l* in isolation, where neurons are connected only via (non-local) inhibitory chemical synapses in a ring network topology. Specifically, we consider Equation (1) with *n*_*l,e*_ = 8, κ_*l,c*_ ≠ 0 and κ_*m,e*_ = κ_*m,c*_ = κ_*l,e*_ = 0. Figure 5 shows the variation of *R*_*T*_ against the noise amplitude σ_*l*_ for this layer. We also fixed ε = 0.0005 ≪ 1 so that Equation (8) can be satisfied in a weak noise limit σ_*l*_ → 0, leading to the occurrence of SISR. We shall now mainly compare the enhancement of SISR in layer *l* for two situations, i.e., when the neurons are locally connected via time-delayed electrical synapses (see Figure 4) and when the neurons are non-locally connected via time-delayed inhibitory chemical synapses (see Figure 5).

The first observation is that longer inhibitory time delays enhance SISR, while longer electrical time delays deteriorate SISR. However, similarly to electric time delays, chemical time delays (τ_*l,c*_) have a strong effect on SISR only for stronger chemical synaptic strength (κ_*l,c*_). In Figure 4A, the electrical synaptic strength is weak (κ_*l,e*_ = 0.1). Even though the interval of the noise amplitude, for which a pronounced SISR occurs (as indicated by the very low values of *R*_*T*_), shrinks on the left bound with increasing time delay, the low values of *R*_*T*_ within that interval remain unchanged (≈ 0.015). Similarly, results in Figure 5A with the same weak inhibitory synaptic strength (κ_*l,c*_ = 0.1) show that changing the chemical time delays does also not affect the low and constant values of *R*_*T*_ ≈ 0.014 (indicating an optimized SISR). In contrast, however, the lower bound of the noise interval with optimal SISR remains independent of varying levels of time delay. Thus, for weak synaptic strength and for increasing synaptic time delays, inhibitory chemical synapses outperform electrical synapses in optimizing SISR, in the sense that the former allow for a wider range of noise amplitudes for which *R*_*T*_ remains low.

In Figure 5B, where a large inhibitory chemical synaptic strength (κ_*l,c*_ = 1.0) is considered, time delays can have significant effect on SISR, and this is in contrast to Figure 5A, where κ_*l,c*_ is weak. In Figure 5B, increasing the chemical time delay enhances SISR by lowering the minimum value of *R*_*T*_. In comparison to Figure 5A, the noise interval for which SISR is optimal has shrunk on both sides. The reason for this shrinking on both ends of the optimal noise interval is essentially the same as for the case where the strength of electrical synapses is varied (see Figures 4B,D). In Figure 5B, we have *R*_*T*_min__ = 0.29 at $\sigma_{l}=3.7\times 10^{-5}$ for τ_*l,c*_ = 5.0; *R*_*T*_min__ = 0.21 at $\sigma_{l}=3.7\times 10^{-5}$ for τ_*l,c*_ = 10.0; *R*_*T*_min__ = 0.17 at $\sigma_{l}=4.6\times 10^{-5}$ for τ_*l,c*_ = 15.0; *R*_*T*_min__ = 0.15 at $\sigma_{l}=4.6\times 10^{-5}$ for τ_*l,c*_ = 20.0; *R*_*T*_min__ = 0.12 at $\sigma_{l}=3.7\times 10^{-5}$ for τ_*l,c*_ = 25.0. However, the deteriorating effects of electrical time delays on SISR is more pronounced than those of the chemical time delays at the same synaptic strength (κ_*l,e*_ = κ_*l,c*_ = 1.0) (see Figures 4B, 5B. This confirms that chemical synapses are better at optimizing SISR than electrical synapses, not only because they allow for a wider range of noise amplitude in which optimal SISR may occur but also for the occurrence of a more enhanced SISR, as indicated by the relatively lower values of *R*_*T*_ at long time delays.

In Figures 5C,D, we investigate the effects of chemical synaptic strength in a short and long time delay regime. Irrespective of the time delay regime, the stronger the chemical synaptic strength is, the more deteriorated SISR is. The reason behind this behavior is the same as the one given for the case of electrical synapses. That is, as the chemical synaptic strength κ_*l,c*_ becomes larger, the energy barriers $△U_{i}^{lc}\left(w_{l,i}\right)$ and $△U_{i}^{rc}\left(w_{l,i}\right)$ become deeper (see Figure 3). However, now a stronger noise amplitude is required to jump over the deep energy barriers and induce spiking, and this strong noise amplitude destroys the coherence of the spiking (by violating the conditions in Equation (8) requiring σ_*l*_ → 0) and hence deteriorates SISR.

The deterioration of SISR by stronger chemical synaptic strengths is also observed with stronger electrical synaptic strength. However, for short synaptic time delay regimes (τ_*l,e*_ = 1.0 = τ_*l,c*_, see Figures 4C, 5C), we notice the following difference for both synaptic types: When the time delay is relatively short, electrical synapses optimize SISR compared to chemical synapses as the synaptic strength is weakened. We see in Figure 5C with τ_*l,c*_ = 1.0 that SISR is destroyed as the κ_*l,c*_ increases, whereas in Figure 4C with τ_*l,e*_ = 1.0, SISR remains enhanced as κ_*l,e*_ increases. This means that an electrical synapse is a better means than a chemical synapse in optimizing SISR at very short time delays, irrespective of the synaptic strengths, while a chemical synapse is better than an electrical synapse at very long time delays, irrespective of the synaptic strengths.

In Figure 5, the reason for the deterioration of SISR with decreasing time delays could be inferred from the reason given for the deterioration of SISR with increasing synaptic strength. That is, shortening the chemical synaptic time delays increases the depth of the chemical energy barrier functions given in Equation (9). This will in turn demand larger noise amplitude to jump over deep energies barriers to induce spiking with no coherence and hence very poor SISR, as seen, for example, from the red curve in Figure 5C. Here, we see that rare spiking can be induced only when the noise $\sigma_{l}\geq 10^{-4}$ as *R*_*T*_ stays high with increasing noise amplitude σ_*l*_. However, from conditions in Equation (8), SISR requires σ_*l*_ → 0, which implies that increasing the noise would not improve SISR. We can see from the red curve in Figure 5C that a minimum value of *R*_*T*_min__ ≈ 0.71, already indicating a very poor SISR, occurs at a relatively large noise amplitude of σ_*l*_ = 0.18. Below, we shall focus on the enhancement of this very poor SISR in layer *l* = 2 (for κ_2,*c*_ = τ_2,*c*_ = 1.0; see also red curve in Figure 5C) by using various multiplexing configurations, where layer *l* = 1 already exhibits enhanced SISR in isolation.

## 6. Multiplexing and Optimization of SISR

We now address the following questions: (1) Is an optimization of SISR based on the multiplexing of layers possible? (2) Which synaptic multiplexing configuration is the best optimizer of SISR? To answer these two questions, we configure the synaptic strength and time delay of one layer (say layer 1) such that SISR is optimal in this layer. The corresponding parameters of layer 2 are configured such that SISR is non-existent in this layer. Then, we connect these two layers in a multiplex fashion (see Figure 1) in six different multiplexing configurations.

In the first three configurations, the two layers of the multiplex network each consist of neurons that are intra-connected by only electrical synapses (κ_1,*e*_, τ_1,*e*_) and (κ_2,*e*_, τ_2,*e*_), and are inter-connected (multiplexed) by (i) electrical synapses (κ_*m,e*_, τ_*m,e*_), (ii) inhibitory chemical synapses (κ_*m,c*_, τ_*m,c*_), and (iii) excitatory chemical synapses (κ_*m,c*_, τ_*m,c*_).

In the next three configurations, we use the same three synaptic multiplexing configurations of two layers, each consisting of neurons that are intra-connected by only inhibitory chemical synapses. We do not consider excitatory chemical synapses for intra-connectivity because this type of synapse induces coherent spiking activities even in the absence of noise, which is not a requirement for SISR. On the other hand, we use excitatory chemical synapses in the inter-layer connections (multiplexing) Because, for some synaptic strengths and time delays, the multiplex network remains in the excitable regime in the absence of noise—a requirement for observing SISR. However, we also have situations in which the multiplexing excitatory chemical synapses strengthen the excitable regime of the network by making the homogeneous fixed point more stable, thereby requiring very large noise amplitudes to have a chance of inducing a spike. Large noise amplitudes, however, violate the conditions necessary for the occurrence of SISR. We shall therefore also avoid such regimes and stay in the excitable regimes where vanishingly small noise amplitudes have a non-zero probability of inducing at least a spike in the large time interval we considered in our simulations.

In the following numerical simulations, we have ensured that the multiplex network stays in the excitable regime by checking that all the synaptic strengths and time delays of the multiplexing synapses are such that no self-sustained spiking activity occurs in the absence of noise. It is worth mentioning here that the optimization of SISR based on the multiplexing approach appears not to be feasible in a network of mixed layer type, i.e., consisting of an electrical layer multiplexed to a chemical layer. We investigated all the possible configurations of mixed layered networks, i.e., those with electrical, inhibitory, and excitatory chemical multiplexing. Extensive numerical simulations (not shown) clearly indicated that the optimization of SISR, for the ranges of the multiplexing synaptic strengths and time delays considered, was not possible in mixed layered networks. For this reason, we only discuss the layered networks that display the capability of optimizing SISR and compare the optimization abilities of various multiplexing connections between two electrical layers and then between two inhibitory chemical layers.

### 6.1. Multiplexing of Electrical Layers

**Figure 6:**
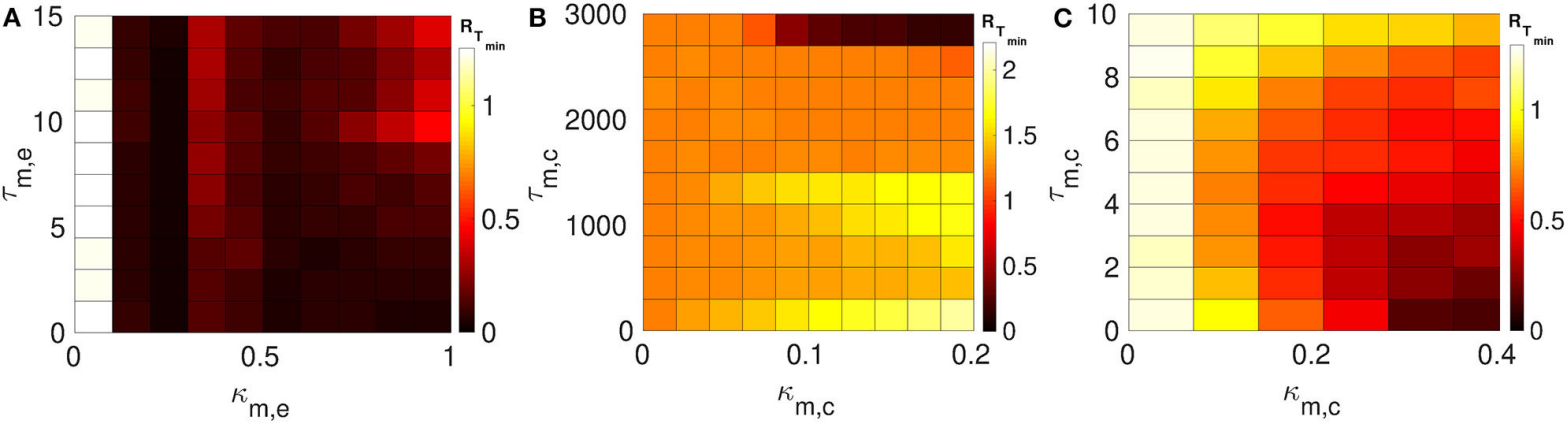
Color-coded minimum coefficient of variation (*R*_*T*_min__) of layer 2 as a function of the multiplexing parameters. Both layers 1 and 2 are intra-connected by electrical synapses. **(A)** Shows the enhancement performances of the electrical multiplexing (κ_*m,e*_, τ_*m,e*_). For optimized SISR in layer 2, we need either a shorter τ_*m,e*_ and stronger κ_*m,e*_; or a longer τ_*m,e*_ and a weaker κ_*m,e*_. **(B)** Shows the enhancement performances of inhibitory chemical multiplexing (κ_*m,c*_, τ_*m,c*_). For optimized SISR in layer 2, we need a stronger κ_*m,c*_ and a very long τ_*m,c*_. **(C)** Shows the enhancement performances of excitatory chemical multiplexing (κ_*m,c*_, τ_*m,c*_). For optimized SISR in layer 2 we need very short τ_*m,c*_ and stronger κ_*m,c*_. Parameters for layer 1, we need *N* = 25, *n*_1,*e*_ = 1, β = 0.75, ε = 0.0005, α = 0.5, κ_1,*e*_ = 0.1, and τ_1,*e*_ = 1.0. Parameters for layer 2, we need *N* = 25, *n*_2,*e*_ = 1, β = 0.75, ε = 0.0005, α = 0.5, κ_2,*e*_ = 1.0, and τ_2,*e*_ = 10.0.

We consider layer 1 and layer 2 in which neurons are electrically coupled only. We choose the synaptic parameters (κ_1,*e*_, τ_1,*e*_) of layer 1 such that SISR is pronounced, i.e., we choose a weak synaptic strength κ_1,*e*_ = 0.1 and a short synaptic time delay τ_1,*e*_ = 1.0 (green *R*_*T*_-curve Figure 4C with *R*_*T*_min__ = 0.015). For layer 2, we set the synaptic parameters such that SISR is non-existent, i.e., we choose a strong synaptic strength κ_1,*e*_ = 1.0 and a long synaptic time delay τ_2,*e*_ = 10.0 (red *R*_*T*_-curve Figure 4D with *R*_*T*_min__ = 1.24). These two layers are then coupled in a multiplex network as shown in Figure 1A. The multiplexing introduces two other parameters—the multiplexing synaptic strengths {κ_*m,e*_, κ_*m,c*_} and their corresponding time delays {τ_*m,e*_, τ_*m,c*_}. Figure 6 shows the color-coded minimum values of the coefficient of variation *R*_*T*_min__ of layer 2 as a function of the multiplexing parameters for the three multiplexing configurations considered.

In Figure 6A, the multiplexing between the two layers is mediated by electrical synapses with parameters (κ_*m,e*_, τ_*m,e*_). We can clearly see that even very weak multiplexing κ_*m,e*_ ≥ 0.1, particularly at short time delays τ_*m,e*_ ≤ 9.5, can induce a very pronounced SISR in layer 2 (where SISR was non-existent in isolation) as indicated by the dark red color corresponding to very low values of *R*_*T*_min__. In the region τ_*m,e*_ ≤ 9.5, stronger multiplexing strengths push *R*_*T*_min__ to even lower values as indicated by the darker red color, thus optimizing SISR in layer 2. However, as the multiplexing time delay becomes longer τ_*m,e*_ > 9.5, this time delay starts to dominate the control of SISR. As the time delay τ_*m,e*_ > 9.5 increases, SISR progressively deteriorates and the effect of strong multiplexing is reversed, i.e., the stronger κ_*m,e*_ is, the more SISR deteriorates, as indicated by the change of color of *R*_*T*_min__ from dark red to light red. While in this same region, i.e., τ_*m,e*_ > 9.5, weaker multiplexing optimize SISR better than strong ones, as seen in the region bounded by τ_*m,e*_ ∈ [9.5, 15.0] and κ_*m,e*_ ∈ [0.1, 1.0] with a dark red color.

In Figure 6B, the multiplexing between the two electrical layers is mediated by inhibitory chemical synapses with parameters (κ_*m,c*_, τ_*m,c*_). We notice, in contrast to Figure 6A, that the multiplexing inhibitory chemical synaptic strength takes a maximum value of κ_*m,c*_ = 0.2, i.e., it stays in the weak multiplexing regime and the time delay goes up to the very large value of τ_*m,c*_ = 3, 000. As already pointed out, we always want the network to stay in the excitable regime such that self-sustained oscillations do not arise due to bifurcations. In this multiplexing configuration, values of the inhibitory chemical synaptic strength greater than 0.2 induces oscillations in the absence of noise—for SISR, the system should oscillate coherently due only to the presence of noise and not due to a bifurcation. As we can see in Figure 6B, weak multiplexing inhibitory chemical synaptic strength κ_*m,c*_ ∈ [0.0, 0.2] cannot induce SISR in layer 2 as the values of *R*_*T*_min__ stay very high above 1.0, except at very long multiplexing delays τ_*m,c*_ ≥ 2, 750. It can be observed that for time delays τ_*m,c*_ ≤ 1, 500, stronger multiplexing values, κ_2,*c*_ ≳ 0.1, deteriorate SISR (yellow region) to a larger extent than the weaker values, κ_2,*e*_ ≲ 0.1 (orange region). But when the multiplexing time delay becomes very long, e.g., at τ_*m,c*_ = 3, 000, stronger multiplexing (κ_2,*c*_ ≳ 0.1) induces an optimized (dark red color of *R*_*T*_min__) SISR in layer 2, while weaker multiplexing (κ_2,*c*_ ≲ 0.1) cannot optimize SISR, as indicated by the orange color of *R*_*T*_min__. This means that multiplexing with inhibitory chemical synapses has the opposite effect compared to multiplexing with electrical synapses, in terms of SISR in layer 2. To sum up, stronger κ_*m,c*_ means poorer SISR at shorter τ_*m,c*_ but better SISR at longer τ_*m,c*_; the opposite is also true, as stronger κ_*m,e*_ means better SISR at shorter τ_*m,e*_, but poorer SISR at longer τ_*m,e*_.

In Figure 6C, the multiplexing between the two electrical layers is mediated by excitatory chemical synapses with parameters (κ_*m,c*_, τ_*m,c*_). First, we notice the range of the synaptic strength and the time delay. For κ_*m,c*_ > 0.4, the excitability of the network becomes so strong that even large noise amplitudes (SISR requires vanishingly small noise) are not longer capable of inducing a spike in the large time interval considered. In contrast to weak multiplexing electrical synapses in Figure 6A, weak multiplexing excitatory chemical synapses are incapable of inducing SISR in layer 2. In Figure 6C, for a weak multiplexing κ_*m,c*_ ∈ [0.0, 0.28], *R*_*T*_min__ remains high with the lowest value above 0.5 (as indicated by the white, yellow, orange, and light red colors of *R*_*T*_min__) for all of the time delays considered. This inability of weak excitatory chemical multiplexing to optimize SISR in layer 2 is similar to that of weak inhibitory chemical multiplexing in Figure 6B. However, for an intermediate excitatory chemical multiplexing, i.e., κ_*m,c*_ ∈ [0.28, 0.4], an optimized SISR is induced in layer 2 (just like with intermediate electrical multiplexing in Figure 6A), but only at very short time delays τ_*m,c*_ ∈ [0.0, 2.0], where *R*_*T*_min__ assumes low values corresponding to the dark red colors of *R*_*T*_min__. And the main difference between inhibitory chemical multiplexing and excitatory chemical multiplexing is in terms of their time delays. While inhibitory chemical multiplexing requires extremely long time delay (τ_*m,c*_ ≥ 2, 750) to optimize SISR in layer 2, excitatory chemical multiplexing requires extremely short time delays (τ_*m,c*_ ∈ [0.0, 2.0]) for the optimization.

### 6.2. Multiplexing of Inhibitory Chemical Layers

**Figure 7:**
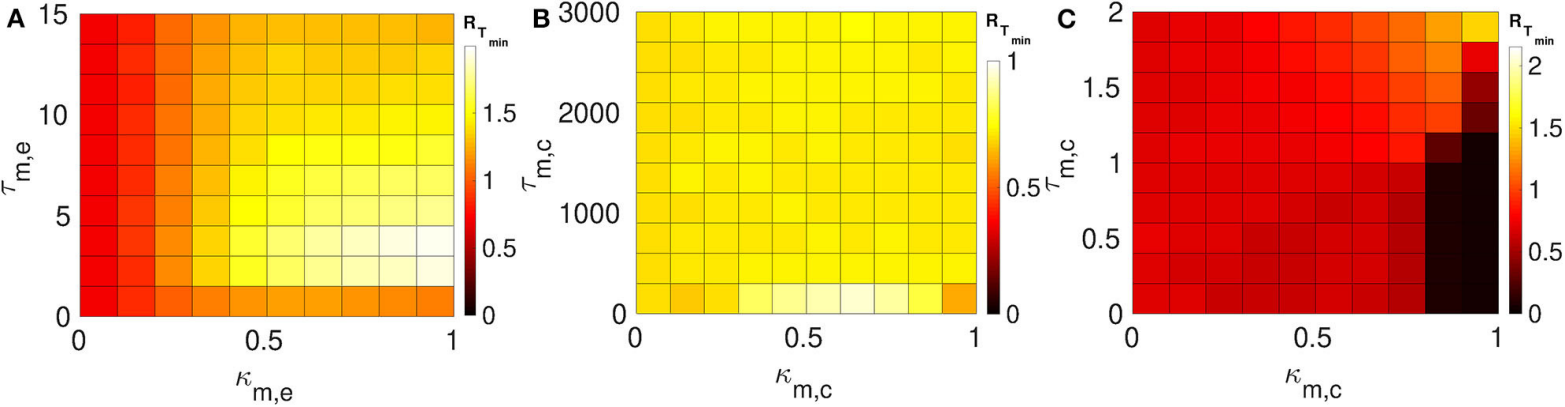
Color-coded minimum coefficient of variation (*R*_*T*_min__) of layer 2 against multiplexing parameters. Each of layer 1 and layer 2 is intra-connected by inhibitory chemical synapses. **(A)** Shows the enhancement performances of the electrical multiplexing (κ_*m,e*_, τ_*m,e*_). Here, we observe that electrical multiplexing cannot optimize SISR in layer 2, especially at stronger multiplexing. **(B)** Shows the enhancement performances of the inhibitory chemical multiplexing (κ_*m,c*_, τ_*m,c*_). In this case, the enhancement of SISR is even worse than in **(A)**, especially at intermediate multiplexing strengths and short time delays. **(C)** Shows the enhancement performances of the excitatory chemical multiplexing (κ_*m,c*_, τ_*m,c*_). Here, an enhancement is possible. An optimized SISR (*R*_*T*_min__ ≈ 0.03) emerging at strong excitatory chemical synapses (κ_*m,c*_ ≥ 0.8) with short time delays (τ_*m,c*_ ≤ 1.2). Parameters of layer 1: *N* = 25, *n*_1,*c*_ = 8, β = 0.75, ε = 0.0005, α = 0.5, κ_1*c*_ = 0.1, τ_1,*c*_ = 25.0. Parameters of layer 2: *N* = 25, *n*_*l,c*_ = 8, β = 0.75, ε = 0.0005, α = 0.5, κ_*l,c*_ = 1.0, τ_*l,c*_ = 1.0.

Here we consider layers 1 and 2 in which neurons are coupled only via inhibitory chemical synapses. We set the synaptic parameters (κ_1,*c*_, τ_1,*c*_) of layer 1 such that SISR is pronounced, i.e., we choose a weak synaptic strength κ_1,*c*_ = 0.1 and a long synaptic time delay τ_1,*c*_ = 25.0, see the green *R*_*T*_-curve Figure 5D with *R*_*T*_min__ = 0.015. For layer 2, we set the synaptic parameters such that SISR is very poor, i.e., we choose a strong synaptic strength κ_1,*c*_ = 1.0 and a short synaptic time delay τ_2,*c*_ = 1.0, see the red *R*_*T*_-curve Figure 5C with *R*_*T*_min__ = 0.71. These two layers are then coupled in a multiplex network as shown in Figure 1B. The multiplexing introduces two other parameters—the multiplexing synaptic strengths {κ_*m,e*_, κ_*m,c*_} and their corresponding time delays {τ_*m,e*_, τ_*m,c*_}. Figure 7 shows the color-coded minimum values of the coefficient of variation *R*_*T*_min__ of layer 2 as a function of the multiplexing parameters for the three multiplexing configurations considered.

In Figure 7A, the multiplexing between the two inhibitory chemical layers is mediated by electrical synapses with parameters (κ_*m,e*_, τ_*m,e*_). It is observed that electrical multiplexing cannot at all optimize SISR in layer 2 as indicated by the very high values of *R*_*T*_min__ in the entire κ_*m,e*_ − τ_*m,e*_ parameter space. Comparing Figure 6A and Figure 7A, we can conclude that electrical multiplexing become good optimizers of SISR only when the multiplexed layers are both intra-connected by electrical synapses. In particular, we observe that, while a strong multiplexing κ_*m,e*_ with a short delay τ_*m,e*_ of layers intra-connected by electrical synapses optimizes SISR in layer 2, (see Figure 6A), a strong multiplexing κ_*m,e*_ with a short delay τ_*m,e*_ of layers intra-connected by inhibitory chemical synapses makes SISR rather worse (*R*_*T*_min__ ≥ 1.0, see Figure 7A) in layer 2 than when this layer is in isolation (*R*_*T*_min__ = 0.71).

In Figure 7B, the multiplexing between the two inhibitory chemical layers is mediated by inhibitory chemical synapses with parameters (κ_*m,c*_, τ_*m,c*_). In this multiplexing configuration, an optimization of SISR in layer 2 is impossible as well, especially at intermediate multiplexing strengths and short time delays, where the *R*_*T*_min__ assumes an even larger value (*R*_*T*_min__ ≈ 0.9) than layer 2 in isolation (*R*_*T*_min__ = 0.71). Moreover, even very long multiplexing time delays, as in the case of electrical layers multiplexed by inhibitory chemical synapses (see Figure 6B), cannot optimize SISR in layer 2 irrespective of the multiplexing strength. Thus, we conclude that multiplexing inhibitory chemical synapses is generally a bad optimizer of SISR in layers intra-connected by either chemical synapses or electrical synapses. However, recall that inhibitory chemical synapses can be very good optimizers of SISR within a layer (see Figure 5).

In Figure 7C, the multiplexing between the two inhibitory chemical layers is mediated by excitatory chemical synapses with parameters (κ_*m,c*_, τ_*m,c*_). Note that the range of multiplexing time delay we considered is very short, i.e., τ_*m,c*_ ∈ [0.0, 2.0]. For τ_*m,c*_ > 2.0, excitatory chemical multiplexing induces self-sustained oscillations in the absence of noise—a regime not required for SISR. In contrast to electrical and inhibitory chemical multiplexing of layers intra-connected with chemical synapses (see Figures 7A,B), excitatory chemical multiplexing of such layers can perform extremely well at optimizing SISR. However, this capability for very strong optimization is only possible at strong excitatory chemical multiplexing (κ_*m,c*_ ≥ 0.8) and very short time delays (τ_2,*c*_ ∈ [0.0, 1.2]) with *R*_*T*_min__ ≈ 0.03. This implies that excitatory chemical synapses, as a multiplexing synapse, could play more important functional roles (than electrical and inhibitory chemical synapses) in neural information processing due to SISR in multiplexed layers intra-connected by inhibitory chemical synapses.

## 7. Conclusion

We have investigated the effects of electrical and chemical synaptic couplings on the noise-induced phenomenon of SISR in isolated layers as well as in multiplexed layer networks of the FHN neuron model in the excitable regime. We have presented the analytic conditions necessary for SISR to occur in isolated layers with neurons connected either via electrical or inhibitory chemical synapses. From these analytic conditions, we have also obtained the minimum and maximum synaptic noise amplitude required for the occurrence of SISR in isolated layers.

Numerical computations indicate that, in an isolated layer, the weaker the electrical synaptic strength and the shorter the corresponding synaptic time delay are, the more enhanced SISR is. However, the deteriorating effect of stronger electrical synaptic couplings is significant only at longer time delays and vice versa. On the other hand, in an isolated layer with inhibitory chemical synapses, weaker inhibitory chemical synaptic couplings just like their weaker electrical counterparts enhance SISR. Moreover, the longer the synaptic time delay is, the more enhanced SISR is—in contrast to isolated layers with electrical synapses. The enhancing effect of the longer synaptic time delays in isolated layers with inhibitory chemical synapses becomes significant only at stronger synaptic strengths. Furthermore, it is also found that at very short time delays and irrespective of the synaptic strengths, electrical synapses are better optimizers of SISR than chemical synapses. Meanwhile, at very long time delays, and irrespective of the synaptic strengths, chemical synapses are a better optimizers of SISR than electrical synapses. The expressions of electrical and chemical interaction potentials together with the minimum and maximum values of the noise amplitude within which an optimized SISR can occur are used to provide a theoretical explanation of the above effects.

After identifying the electrical and chemical synaptic strengths and time delays that destroy (or optimize) SISR in an isolated layer, we proceeded with identifying multiplexing configurations between the two layers that would optimize SISR in the second layer where SISR would be very poor or non-existent in isolation. For this identification, the synaptic parameters of one layer is configured such that SISR is optimal and this layer is multiplexed with a second layer where synaptic parameters are such that SISR is very poor or even non-existent. We then investigated which multiplexing connection (i,e., electrical, inhibitory chemical, or excitatory chemical synapses) is a better optimizer of SISR in the second layer.

In the first optimization configuration, we were interested in optimizing SISR in an electrically coupled layer (i.e., a layer where neurons are coupled only via electrical synapses) by multiplexing this layer with another electrically coupled layer. We found that even weak multiplexing with electrical synaptic connections may optimize SISR in the layer where SISR was even absent in isolation. However, the longer the multiplexing electrical synaptic time delay is, the less efficient this configuration becomes in optimizing SISR. In a second scenario, the multiplexing connection was mediated by inhibitory chemical synapses between these electrical layers. Here, we found that only very long multiplexing inhibitory chemical synaptic time delays at weak (but not too weak) synaptic strength may optimize SISR in the layer where it was non-existent in isolation. And in the third scenario, the multiplexing connection was mediated by excitatory chemical synapses between these electrical layers. It is found that only very short multiplexing excitatory chemical time delays at intermediate synaptic strengths can optimize SISR in the layer where the phenomenon is non-existent in isolation.

In the second optimization configuration, we were interested in optimizing SISR in an (inhibitory) chemically coupled layer (i.e., a layer where neurons are coupled only via inhibitory chemical synapses) by multiplexing this layer with another (inhibitory) chemically coupled layer. Here it is found that the optimization of SISR based multiplexing between chemical layers does work equally well as in the case of the multiplexing between electrical layers. Multiplexing of the chemical layers by electrical synapses and inhibitory chemical synapses cannot optimize SISR at all in the chemical layer, where in isolation SIRS is otherwise very poor. We found that only multiplexing excitatory chemical synapses (using a strong synaptic coupling and short time delay regime) can optimize SISR in the chemical layer, whereas SISR in isolation is very poor.

Comparing the first and the second optimization configurations of SISR, we conclude that the optimization of SISR is generally better in layers with electrically coupled neurons rather than with chemically coupled neurons, provided that multiplexing connections between the layers are either electrical or inhibitory chemical synapses. Vice versa, optimization of SISR is generally better in layers with (inhibitory) chemically coupled neurons than with electrically coupled neurons, when multiplexing connections between the layers are excitatory chemical synapses.

The manipulation of chemical and electrical patterns in the brain has become more accessible, either via drugs that cross the blood brain barrier, via electrical stimulation delivered through electrodes implanted in the brain, or via light delivered through optical fibers selectively exciting genetically manipulated neurons (Runnova et al., 2016; De Domenico, 2017; Andreev et al., 2018); however, the manipulation of the functional connectivity seems to be a more difficult goal to achieve. Our approach of modeling multi-layer networks in combination with stochastic dynamics offers a novel perspective on the modeling of the brain's structural and functional connectivity. We therefore expect that our findings could provide promising applications in controlling synaptic connections to optimize neural information (generated by noise-induced phenomena like SISR and CR) processing in experiments, surgery involving brain networks stimulation, and in designing networks of artificial neural circuits to optimize information processing via SISR (Eberhardt et al., 1989; Moopenn et al., 1989, 1990).

Interesting future research directions on the topic would be to investigate the optimization performances of electrical and chemical synapses in other intra-layer topologies like small-world network, scale-free network, and random network; and other inter-layer topologies like the multiplex topology in which neurons in one layer are connected (randomly) to more than one neuron in the other layer.

## Data Availability Statement

The raw data supporting the conclusions of this article will be made available by the authors, without undue reservation.

## Author Contributions

MY designed the study, conducted the analysis and simulations, and wrote the manuscript. PH conducted the analysis and wrote the manuscript. EM conducted the analysis and wrote the manuscript. All authors contributed to the article and approved the submitted version.

## Conflict of Interest

The authors declare that the research was conducted in the absence of any commercial or financial relationships that could be construed as a potential conflict of interest.
